# Impact of an analytical treatment interruption on partners and family members of trial participants in Durban, South Africa: a qualitative study

**DOI:** 10.3389/fpubh.2025.1662141

**Published:** 2025-12-15

**Authors:** Krista L. Dong, Mzwakhe Wiseman Ngcobo, Ntombifuthi Langa, Ayanda Zulu, Luyanda Maphalala, Vanessa Pillay, Maud Mthembu, Whitney Tran, Rachel Lau, Annie Miall, Deborah Mindry, Ali Ahmed, Thumbi Ndung’u, Karine Dubé

**Affiliations:** 1 Ragon Institute of Massachusetts General Hospital (MGH), Massachusetts Institute of Technology (MIT) and Harvard, Cambridge, MA, United States; 2 Massachusetts General Hospital (MGH), Boston, MA, United States; 3 Harvard Medical School, Cambridge, MA, United States; 4 Integration of TB in Education and Care for HIV/AIDS (ITEACH), Harry Gwala Regional Hospital Formerly Edendale Hospital, Pietermaritzburg, South Africa; 5 Females Rising Through Education, Support and Health (FRESH), Durban, South Africa; 6 Department of Social Work, School of Applied Human Sciences, University of Kwa-Zulu Natal (UKZN), Durban, South Africa; 7 Division of Infectious Diseases and Global Public Health, School of Medicine, University of California San Diego (UCSD), La Jolla, CA, United States; 8 Center for Gender and Health Justice, University of California Global Health Institute, Oakland, CA, United States; 9 HIV Pathogenesis Programme (HPP), The Doris Duke Medical Research Institute, Durban, South Africa; 10 Africa Health Research Institute (AHRI), Nelson R. Mandela School of Medicine, Durban, South Africa; 11 Division of Infection and Immunity, University College London, London, United Kingdom

**Keywords:** socio-behavioral research, HIV cure research, analytical treatment interruptions, loved ones, implementation, psychosocial support, HIV prevention (PrEP), South Africa

## Abstract

**Background:**

HIV cure-related research often include analytical treatment interruptions (ATIs), which are monitored pauses in antiretroviral therapy (ART) to determine whether interventions can stimulate viral control without ART. While ATIs have been conducted in high-income countries, scale-up in low-income HIV high-burden countries in sub-Saharan Africa raise unique ethical, social, and practical challenges. HIV cure-related trials focus on participants without considering the experiences of partners and family members. Here, we explore the perspectives of partners and family members, including their emotional, relational, and mental health, during an ATI-inclusive HIV cure trial conducted in Durban, South Africa.

**Methods:**

We conducted a qualitative socio-behavioral study to explore the experiences of close contacts of ATI trial participants. Between November 2022 and June 2024, we interviewed partners and family members referred by trial participants. Interviews explored understanding of the trial, emotional and relational impacts, and concerns about ATIs. The interviews were conducted in English and/or *isi*Zulu, transcribed, translated, and analyzed using content analysis to identify themes related to partner protections, trial communication, and psychosocial support.

**Results:**

Ten participants comprised five male partners (two living with HIV, three without) and five female family members (two mothers, two sisters, one cousin). Most had limited knowledge of HIV cure research but expressed hope for its advancements alongside concerns about ART discontinuation and viral rebound. Partners without HIV valued pre-exposure prophylaxis (PrEP) but reported inconsistent use, while partners with HIV feared re-infection during viral rebound. Mothers expressed concerns about ATIs, while sisters sought clearer information. Participants recommended improved communication, partner protections and psychosocial support, while acknowledging the trial’s scientific importance.

**Conclusion:**

ATI-inclusive HIV cure trials affect participants and their close social networks comprised of family members and intimate partners. Ethical trials are responsible for ensuring the safety of participants and other impacted groups. Family and partners were a critical source of support for trial participants, but have been underutilized in ATI-inclusive trials. Leveraging this existing support network in future ATI trials may improve safety while facilitating recruitment, willingness to discontinue ART during ATIs, prevent early ATI discontinuation, support adherence to frequent visits and sampling requirements, improving overall trial success.

## Introduction

Global human immunodeficiency virus (HIV) incidence has decreased significantly since its peak in 1995 (1), largely due to the scale up of public health measures in the last two decades, including increased access to effective antiretroviral treatment (ART) (2). However, HIV incidence and prevalence in sub-Saharan South Africa remains high (3). Notably, young women aged 15–24 years are at highest risk of HIV acquisition (3–5) due to a combination of factors, including biological susceptibility (6), intimate partner violence (IPV) (7–9), poverty (10, 11), and stigma (12, 13). Curbing the HIV epidemic through ART and pre-exposure prophylaxis (PrEP) is dependent upon uptake and adherence (14, 15). Moreover, there are challenges of emerging drug resistance, drug side effects and persistent immune dysfunction despite fully suppressive ART. These concerns have prompted a global push to develop HIV cure interventions capable of inducing sustained HIV control in the absence of ART (16–18).

The Females Rising through Education, Support, and Health (FRESH) program, located in the Umlazi township, in Durban, South Africa, aims to reduce the risk of HIV acquisition among young women by providing a socio-economic empowerment, job and life skills program that includes an HIV prevention component and access to PrEP (19). Groups of up to 30 young women, aged 18–24 years, participate in a nine-month observational study that includes blood and tissue sampling, combined with twice-weekly screening by ribonucleic acid (RNA) testing to detect new HIV infections as early as possible (20). Women who acquire HIV are initiated on immediate ART and followed longitudinally. From 2020, the FRESH site began implementing interventional clinical trials (21–23). In 2022, FRESH initiated an HIV cure trial to assess the ability of a regimen of dual broadly neutralizing antibodies (bNAbs) and a toll-like receptor 7 (TLR-7) agonist to induce post-intervention control (24). The trial included an analytical treatment interruption (ATI) which is a monitored pause in ART, based on pre-determined criteria to assess whether the intervention may induce viral control in the absence of treatment (25, 26). While ATIs have been utilized in European and United States (U.S.) research contexts, conducting an ATI-inclusive trial at FRESH represents a milestone for trial implementation in sub-Saharan Africa, demonstrating the feasibility in an HIV high-burden research limited setting.

Given the risk of viral rebound during an ATI (25–30), concerns surrounding potential participant harms as well as partner protections and disclosure have emerged as focal points in the socio-behavioral and ethics literature on HIV cure research (31–35). It is crucial to acknowledge the potential of participant harms and risk of HIV transmission to sex partners of ATI participants, particularly given that two cases of unintentional HIV transmission have been documented in the context of ATI-inclusive clinical trials (36, 37). In response, investigators have sought to better understand the challenges ATI trial participants face related to protecting their partners without HIV (38). Because partners are not enrolled in the ATI trial, few studies have assessed their experiences during ATIs. Further, trial participants may experience clinical and psychosocial impacts, and partners and family members may be affected. One qualitative socio-behavioral study conducted among ten HIV serodifferent couples in the U.S. revealed that partners would be significantly affected by an ATI, despite not being enrolled in the trial (39–41). Additionally, the relevance of dyadic coping between partners in decision-making underscores the importance of assessing relationship dynamics (42). Understanding how partners view and experience ATI trials can help improve how trials approach participant support, partner protections and disclosure.

HIV cure-related trials affect participants and their partners, as well as family members and loved ones (43–45). To our knowledge, few studies have assessed how families are affected when one of their members participates in an HIV cure trial. A study conducted in Southern California with next-of-kin/loved ones of people with HIV (PWH) enrolled in HIV cure research showed that loved ones felt responsible for protecting participants from adverse health and social impacts (43, 44), underscoring the importance of better understanding this dynamic. While a few studies involving partners and family members have been conducted in the U.S. (39, 40) and the Netherlands (46), there is a pressing need to gain an understanding of this dynamic in sub-Saharan Africa where plans are underway to conduct multiple ATI-inclusive HIV cure trials. Relationship and power dynamics, as well as risks of social stigma and discrimination, can hinder HIV-serostatus disclosure (47). Incorporating research to assess the experiences of partners and family members during HIV cure trials offers an opportunity to understand how participant needs can be addressed through trial design (48).

Informed by principles of community-based participatory research (CBPR) (49), emphasizing collaborations between researchers and community members, as well as relational theory (50), centering on the importance of relationships embedded within the socio-cultural environment, this study aimed to increase the understanding of how the close contacts of participants in an ATI-inclusive trial perceived and experienced having their loved one participate in an ATI-inclusive HIV cure trial. We chose to included intimate partners and family members in this study because is known that many young women in South Africa live with their families, including extended family; communal living is common due the need for social and economic support (51, 52). Additionally, sex partners significantly influence the decisions of young women, with unequal gender dynamics, social norms, and economic factors contributing to this influence (53). In this study, we sought to determine whether these two important groups were affected by the ATI, and if they influenced the trial participants’ experiences.

## Methods

### Reflexivity

Our socio-behavioral research team was diverse in terms of race, ethnicity, gender, HIV status, national origin, and place of residence. Team members identified as Black/African, Asian, and White. The team included medical doctors, PhD/DrPH-level socio-behavioral scientists, MPH-trained researchers, clinical research staff, and research associates/assistants. All co-authors shared a strong commitment to community-based participatory research (CBPR) and person-centered approaches, with a dedication to amplifying the voices of individuals affected by HIV cure-related trials and to supporting trial participants, their partners, and family members.

Recruitment and informed consent were separate from the clinical trial. Study staff who conducted the interviews were certified HIV counselors trained in Good Clinical Practice (GCP), and fluent in English and *isi*Zulu. They had prior experience conducting socio-behavioral research associated with interventional clinical trials. The study team received training from experienced senior socio-behavioral scientists that covered the study protocol and informed consent procedures; ethical conduct of research; the ATI trial protocol; partner protections counseling content, including condom use and oral pre-exposure prophylaxis (PrEP).

To support rigor and reflexivity, the team held regular debriefs to review field notes, discuss positionality, and address emergent ethical issues. Supervisors also reviewed a subset of early transcripts to provide feedback on interviewing style and adherence to the guide. We maintained reflexive memos throughout the study to document decision-making processes and consider potential influences on interpretation.

### Study setting and Participants

Between November 2022 and June 2024, we conducted in-depth interviews with partners and family members of participants in the ATI-inclusive HIV cure trial conducted at the FRESH clinical research site in Durban, South Africa. We selected a qualitative research design to achieve a deep understanding, gather contextual insights, and maintain flexibility in our methods and probing while prioritizing the perspectives of the individuals involved (54). In designing the socio-behavioral study, we were inspired by principles from relational theory (50), which emphasizes the role of relationships in understanding human behavior and social dynamics. Relational theory suggests that individual perspectives are shaped by interactions with others, including aspects of interconnectedness, power dynamics, and broader social context (50).

Partners and family members of HIV cure trial participants were referred to our study by trial participants. In other words, trial participants acted as gatekeepers for the study. Since only a portion of the trial participants disclosed their HIV status and trial participation to their partners and family members, we used convenience sampling and only interviewed those who expressed interest in participating. Eligible partners and family members were ≥18 years old, able to provide informed consent, and willing to participate in an in-depth interview (IDI). Prior to each IDI, partners and family members received an Institutional Review Board (IRB)-approved fact sheet explaining the parent clinical trial and the integrated socio-behavioral research study engaging partners and family members of trial participants. Participants were informed that the study team was interested in hearing their thoughts and experiences to help improve the design of future ATI-inclusive HIV cure trials.

### Data collection

Following CBPR approaches (49), we developed and pilot-tested the IDI instruments in close collaboration with community advisory board members who provide oversight for the FRESH program. Community members reviewed the document to ensure the content was appropriate and easy to understand. The two IDI guides—one for partners and one for family members—were available in both English and *isi*Zulu, with professionally certified translations. IDI guides covered topics including perceptions, understanding, and knowledge about the FRESH HIV cure trial, social support, and additional recommendations for ATI trials. In addition, partners of FRESH trial participants were asked questions about acceptable partner protection measures during ATIs (see Table 1).

**Table 1:** IRB-approved interview guide—partners and family members of FRESH trial participants (Durban, South Africa—2022–2024).

Interview guide
Section for partners of ATI trial participants
Icebreaker
How are you doing today? Can you please tell me about your relationship with the trial participant?
Partners’ understanding of the clinical trial
What do you know about the clinical trial? What do you understand about the purpose of the clinical trial?
Why do you think your partner decided to participate in the clinical trial?
How do you feel about your partner participating in the clinical trial?
What questions do you have about this clinical trial?
Partners’ perceptions of ATIs
How would you describe what an ATI (pausing ARV drugs) is to someone else?
What is your understanding of why ‘analytical treatment interruptions’ (ATIs) or pausing ARV drugs are included in clinical trials aimed at post-intervention control?
What concerns do you have for your partner during the ‘ATI’ (pausing ARV drugs)?
Do you feel at risk in anyway because your partner is participating in this clinical trial or ATI?
Partner disclosure and acceptable protection measures
Did the research team explain how sexual partners could be affected by your partner’s participation in the clinical trial?
What protection measures from the clinical trial were you informed about? Is there anything the study could do to help protect you and your sex partner from HIV? Are you aware of the pre-exposure prophylaxis (PrEP) referral option available for partners?
How concerned are you about your partner transmitting HIV to you?
Can you please tell us about the method(s) of protection you plan to use to keep yourself safe during the study? Which protection measures are acceptable to you?
What other protection measures do you think clinical trials that involve an ATI (pausing ARV drugs) should be offered?
How do you prefer to talk about protection?
Social support
What role, if any, will you be play toward support of your partner during this clinical trial? During the ATI (pausing ARV drugs)?
Is there any additional support you feel would be helpful for those undergoing the clinical trial?
Additional considerations
Do you have any additional recommendations for the trial team or other researchers designing future similar trials?
Section for family members of ATI trial participants
Icebreaker
How are you doing today? Can you please tell me about your relationship with the trial participant?
Family members’ understanding of the clinical trial
What do you know about the clinical trial? What do you understand about the purpose of the clinical trial?
Why do you think your family member has decided to participate in the clinical trial?
How do you feel about your family member participating in the clinical trial?
What questions do you have about this clinical trial?
Family members’ perceptions of ATIs
How would you describe the ATI (pausing ARV drugs) to someone else?
What is your understanding of why ‘analytical treatment interruptions’ (ATIs) or pausing ARV drugs are included in trials aimed at post-intervention control?
What concerns do you have for your family member during the ‘ATI’ (pausing ARV drugs)?
Do you feel at risk in any way because your family member is participating in this trial or ATI?
Social support
What role, if any, will you be playing toward support of your family member during this trial? During the ATI (pausing ARV drugs)?
Is there any additional support you feel would be helpful for those undergoing the clinical trial?
Additional considerations
Do you have any additional recommendations for the clinical trial team or other researchers designing future similar trials?

After obtaining informed consent, participants completed a brief demographic questionnaire. A trained interviewer (N. L., A. Z.) built rapport with participants and followed the IRB-approved IDI guides. IDIs were conducted in the participant’s preferred language (*isi*Zulu, English, or a combination). All IDIs were audio recorded and transcribed verbatim, while ensuring text was de-identified. Transcripts were then translated into English and securely stored on a data management platform and were not returned to participants for correction. IDIs lasted between 30 and 60 min. Participants received 150 South African rands (approximately 9 U.S. dollars) for their time. Interviewers completed field notes following each interview.

### Data analysis

We employed conventional content analysis (55) to analyze the qualitative data. We read the IDI transcripts multiple times to create a preliminary codebook that included code names, brief descriptors, and examples. A social scientist (K. Du.) conducted data coding using an open coding method and developed the initial emerging coding tree using an inductive method. Two additional study team members (W. T., A. A.) reviewed and enhanced the codes. We identified significant text units, assigned codes, and organized the resulting codes into narratives within each key category (56). We resolved discrepancies through discussion and consensus. We primarily used manual coding to facilitate data management and analysis as well as collaboration between team members. Participants did not provide feedback on the findings.

We followed the Consolidated Criteria for Reporting Qualitative Research (COREQ) checklist for reporting qualitative research results (Supplementary Table S1). We present representative quotes for the main themes in the results section, with additional quotes available in Supplementary Table S2.

### Ethics statement

The University of KwaZulu-Natal (UKZN) Biomedical Research Ethics Committee (BREC) (#00002897/2021) served as the IRB of record. Additionally, the Mass General Brigham (MGB) IRB (protocol #2022P000729) and the University of California San Diego (UCSD) IRB (#806466) reviewed and approved the research. All participants provided written informed consent to participate, and all aspects of the study were conducted in accordance with the Declaration of Helsinki.

## Results

Nineteen of the 20 HIV cure trial participants took part in the socio-behavioral research study and referred 10 partners and family members who agreed to be interviewed. These included five male partners, aged 27–35 years old, all cisgender men. The partners, two living with HIV and three living without, had been involved in relationships with trial participants for 2–13 years. In addition, we enrolled five family members: two sisters, two mothers, and one cousin, all of whom were cisgender women, between 19 and 50 years of age (Table 2). Most had limited prior familiarity with HIV cure research.

**Table 2:** Demographic characteristics of study participants (Durban, South Africa, 2022–2024).

Participant number	Relationship to FRESH participant	Sex and gender	Race and ethnicity	Age (years)	HIV status (if known and disclosed)	Familiarity with the HIV post-intervention trial at FRESH
Boyfriends/partners						
03	Boyfriend/partner (6 years)	Cisgender man	Black/African	35	Without HIV	A little familiar
04	Boyfriend/partner (4 years)	Cisgender man	Black/African	27	Without HIV	General understanding
05	Boyfriend/partner (13 years)	Cisgender man	Black/African	33	With HIV	Not at all
07	Boyfriend/partner (8 years)	Cisgender man	Black/African	32	With HIV	Not at all
08	Boyfriend/partner (2 years)	Cisgender man	Black/African	30	Without HIV	Not at all
Family members						
01	Sister	Cisgender woman	Black/African	23	Unknown	A little familiar
02	Cousin	Cisgender woman	Black/African	39	Unknown	General understanding
06	Mother	Cisgender woman	Black/African	50	Unknown	Not at all
09	Sister	Cisgender woman	Black/African	19	Unknown	Very familiar
10	Mother	Cisgender woman	Black/African	48	Unknown	General understanding

The limited number of interviews reflected nondisclosure of HIV status or trial participation by some index participants, scheduling barriers or lack of time among referrals, declinations, transient partnerships, and unreturned messages.

### General experiences of the HIV cure trial

Partners and family members had different levels of understanding about the clinical trial and its purpose. Many viewed it as an opportunity to advance HIV cure research, expressing hope that it might one day eliminate the need for ART.

*The purpose is to pause HIV treatment from those who take it [study drug(s)], and then there is a new research and new procedures, where they can take the study drugs so that their system can fight viral load without treatment*. – 04 (Partner without HIV)

*What I know since she is here, she told me that you are trying to do a research so that people who are HIV-positive [have HIV] can end up not having to take ARVs.* – 01 (Sister)

Loved ones described relational effects that included greater openness and communication. Several attributed improved dialogue to counseling and supportive interactions at FRESH, which they perceived as fostering emotional and relational healing.

*My relationship with [HIV cure trial participant], I could say it’s alright now, since she started this study [trial], we’ve been able to talk as a sister about everything. I don’t know how to explain but we are now open to each other, we are able to talk about anything… It was because of the ideas she got from here [FRESH], the counselling and talking with people here… that made her to be the person she is.* – 09 (Sister)

Partners and family members expressed a desire for more detailed information about the trial, including the ATI and processes involved in the experimental intervention. They indicated a need for clearer communication to avoid misconceptions about the trial.

Mothers often voiced early concern about pausing treatment during the ATI, then described accepting the decision after receiving additional information and observing close monitoring. They reflected on supporting their daughters’ choices and on the implications of research participation.

*I did have those concerns but there was nothing I could do because, that was the doctor’s [researcher’s] instructions [were to] pause her [ART] while using study drugs. Fortunately, nothing bad happened to her. I was skeptical as I was thinking but they say you take these pills for the rest of your life, now they are saying she must stop. She explained everything to me, and I understood.* – 06 (Mother)

*Yes, I’d say she gets a lot of [benefits from the trial] because she is taught many things. She doesn’t ask me anything like how does 1 and 2 happen. So now she has knowledge. She is the one who tells me how it is going, if you do this and that you will be alright, so that is why I say she’s got knowledge here and I do not have a problem that she is here.* – 10 (Mother)

Partners and family members also expressed a strong sense of resilience in dealing with HIV-related challenges. They found strength in their relationships and supported each other through emotional highs and lows. Participation in the trial was often described as a turning point that encouraged open discussion about health, self-acceptance, and confronting stigma.

Partners and family members perceived that their loved one’s motivations for joining the HIV cure trial involved a desire to advance HIV cure science, learn about their health condition, and strive for self-improvement. Narratives conveyed a sense of optimism regarding the potential to contribute to scientific advancements, with one partner indicating that the participant’s reasons for joining the trial included a desire to be at the forefront of any potential future curative intervention.

*She is a person who likes challenges. She wishes to lead a normal healthy life whilst having this virus. She does not want to face any difficulties in future. If there is cure, she wishes to be the first to use it.* – 08 (Partner without HIV)

The desire to confront challenges and to live a fulfilling life despite a HIV diagnosis was another emerging theme. Partners and family members viewed loved ones as actively seeking solutions through clinical research, reflecting a proactive stance toward their health and the health of future women with HIV.

Partners and family members generally supported their loved ones’ participation in the HIV cure trial, although they expressed both hope and caution. Despite some initial concerns raised, particularly by mothers, the overall experience of family members was that the trial fostered mutual adaptability, resilience, and support.

*I said if she feels it is the right thing to do, she can do it. I do not have a problem with that. I accepted what she is doing… Since she took this opportunity, so far, we are good, we support each other*. – 03 (Partner without HIV)

Partners and family members reported positive feelings about the clinical trial, highlighting improvements in their loved ones’ quality of life and vitality. Many reported perceived weight gain and increased appetite in trial participants, interpreting these changes as signs of improved health. Other perceived benefits included regular clinical monitoring and not having to take ART. One partner believed the trial participant could eventually be free from HIV because of the trial.

*I think already we are moving [a] step forward. She is no longer taking her pills and her viral load is suppressed, that alone is a benefit. If she can continue until it is proven that she no longer has the virus, then that will be a biggest benefit*. – 03 (Partner without HIV)

*I will say she has benefited especially on [the trial]. I can see she has gained weight. I do not want to lie, it is boring even for me to take a [anti-HIV] pill on daily basis. If it is up to me, I would have also joined this study [trial]. If men were also allowed on this study [trial], I would have joined this thing [trial] and go for lymph node procedure and all that … and come back free of taking ARVs [ART]. So that I will live as before, but for her I can see she has gained a lot*. – 05 (Partner with HIV)

However, concerns emerged regarding potential health setbacks, such as a decline in immune function, and added emotional burdens associated with trial participation. A partner with HIV expressed worries about communication difficulties arising because of the trial. Overall, partners and family members expressed trust in the trial and the research team and believed that enrolment in the trial would lead to improved long-term health outcomes for their loved one living with HIV.

Partners and family members expressed a need for information about potential health risks associated with the trial, particularly concerning viral rebound during the ATI. Partners without HIV expressed heightened concern about the risk of HIV acquisition, sought means of protecting themselves and wanted clearer information about the effectiveness of available HIV prevention methods in the event of viral rebound.

*The question I had was what will happen if I decide to stop taking PrEP? I had that concern and they said I can stop anytime I like. That is the only thing that I was worried about, and how [effective] is PrEP to protect if ever my partner rebounds. What I wanted to know is will I be able to stop PrEP anytime without problems? … What will happen to me when my partner rebounds?* – 03 (Partner without HIV)

Partners, mothers, and sisters described an emotional burden stemming from concerns about their loved ones’ health during the trial. Additionally, prior experiences with non-adherence to ART in the community heightened loved ones’ anxiety around pausing ART during the ATI. Contributing to these anxieties was knowledge that ART improves survival for PWH and familiarity with the message that ART adherence is critical.

Partners and family members emphasized the need for simple materials, clarity on where to ask questions, and examples of people who paused treatment during an ATI and sustained viral control.

#### Perceptions and experiences during the ATI

Partners and family members supported the use of ATIs as a means of assessing interventions aimed at post-intervention control. While they had varying levels of understanding about the ATI process, some correctly explained that the ATI was designed to evaluate the effectiveness of experimental interventions and to explore whether HIV could be managed without ongoing ART.

*Yes, because the doctors are still looking for the cure, right? As they [participants] are on pause, they are given study drugs and they [researchers] test them [participants] regularly to see which type can fight this virus. Because if they [researchers] find [a] cure, everyone on this treatment will take cure treatment*. – 06 (Mother)

However, partners and family members were concerned that ATI trials contradicted well-ingrained public health messaging about ART adherence and anticipated challenges explaining ATI to others. They worried that ATIs could lead to misconceptions and societal stigma associated with pausing ART and wanted to have a better understanding of the ATI process, both for their own peace of mind and to enable them to explain an ATI to others.

*In a situation where a person is supposed to be taking treatment, it is not easy for them to understand but I can explain to them in a way that they will understand that just because they are pausing their treatment does not mean they are now negative. But they are still on trial, that’s why they are given doses not just that you are changing your lifestyle and you are no longer HIV positive [have HIV]. But in my own view, it is not easy as they are taught differently in clinic, and here you are telling them another way, so they [broader community] will assume you want to kill them [FRESH participants]* – 10 (Mother)

Overall, partners and family members were optimistic about scientific advancements but concerned about the implications of participating in an ATI and explaining ATI trials to others.

Partners and family members expressed a variety of concerns about the ATI. Their main worries centered on potential adverse health effects of pausing ART on their loved one, including viral rebound, declining health, and potential for increased risk of other illnesses. However, they were reassured by regular clinical monitoring, which helped alleviate these fears.

Partners worried about their partner stopping their ART, which is proven to be safe and effective, and instead having to deal with risks from the ATI and an experimental intervention with unknown risk and benefit. They also specifically highlighted concerns about how the ATI might affect their relationships dynamics, especially those not living with HIV.

*I am concerned of [about] what if she stops in one day, and it [HIV] comes back. Yes, maybe it [HIV] comes back more powerful because we know when she is taking treatment, she will be fine. The treatment [ART] works well, we have known it for some time. Now if we try something new, we do not know the results. I have concerns as I am dating her, if I will get infected [acquire HIV] or must take care of her, while she is struggling about this thing. I do now know my concern will be high thinking about things like her life span, all those things. But I have hope since there are people who succeeded with [the experimental intervention], so in that case I have hope.* – 04 (Partner without HIV)

Likewise, some family members were very involved and concerned about their loved one’s physical and mental health during the ATI.

*If it happens that she gets sick and loses weight she says it’s because she is no longer taking her medication … When she sees herself losing weight, she will say it’s because they made her pause the pills. But I made sure that I see everything she did, how she eats, is she sleeping enough, even though sometimes in the afternoon I will not be in the house because of work, but I will still want to know how she is doing, did she have any destruction? Did she get sick? Because you would see someone is alright on the outside, but on the inside, something is eating her. But she was free, and she did not have any problem, so I also did not have any problem.* – 10 (Mother)

All three partners without HIV recognized the risk of acquiring HIV during ATIs and yet accepted this risk and found some reassurance in HIV prevention methods and open communication with the clinical trial participants. In turn, partners with HIV were concerned about the potential impact of ATIs on their own health and the risk of re-transmitting HIV to the ATIs clinical trial participants, and did not want to interfere with the ATI trial. Partners also expressed worries about how ATIs might affect the health of their children.

*I knew what I was putting [getting] myself into*. – 03 (Partner with HIV)

*When she is not taking her treatment, would the viral load not increase… that risk will put both me and my child at risk … I do not want my child to grow up without her mother. So, my concern is that, when she is not taking her treatment, would the viral load not increase? Sometimes I feel like this is a risk*. – 04 (Partner without HIV)

One partner with HIV expressed anxiety about being blamed by family members if they learned about the ATI, which could be perceived as HIV treatment default. Unlike partners, mothers and sisters did not perceive any risks to themselves.

*In my view, before all this started, I only saw risks, because I thought she will default [on her HIV treatment] or get very sick. Another thing if she gets sick, I will also get sick; also, her family will blame me if she defaults. They will think I am careless; [if] I do not remind her to take her treatment*. – 07 (Partner with HIV)

### HIV transmission risk during the ATI

Partners recognized the importance of informed decision-making and behavioral adjustments during the ATI. They emphasized the significance of protection and maintaining open communication throughout the clinical trial.

Partners without HIV generally understood oral PrEP as an effective HIV preventive measure, but their confidence in using it varied. Some had initial concerns about its effectiveness yet found it helpful for managing their HIV serodifferent sexual relationships. One partner without HIV incorporated oral PrEP into his routine but said that he was not aware that PrEP was available at local clinics until he was informed by the research team. Additionally, partners without HIV expressed reluctance to use condoms because they believed condoms reduced sexual pleasure. Despite this, they recognized the combination of oral PrEP and condom use together was the best means of minimizing HIV acquisition risks. Partners without HIV also noted that abstinence was not a reasonable option for their protection.

*Today I am going to take this treatment [PrEP] to protect me from getting infected [acquiring HIV], and they advised me to use condoms. However, sometimes it becomes difficult to use them. I often feel like I don't want to use a condom, which is normal. This way of taking pills [PrEP] is easier because I feel motivated to take them. I had a discussion with a counsellor about PrEP, and he explained everything to me. I understood everything, and after [the] tests, they confirmed my kidneys and liver are fine. I think I am covered on the PrEP part, and he emphasized the importance of using condoms.* – 03 (Partner without HIV)

*Eish what I am worried about is that if “sengimngcolise umunwe” [slang for marrying her] I will no longer perform sex as “brown dash” [slang for without condom or without protection] on her, this means I will always have to “wear tie” [use protection or wear condom] with her … Okay, what I mean is that we can no longer do it without protection, so I have to always protect her and me.* – 05 (Partner with HIV)

*I am taking it [PrEP] so I am safe, but before I had that concern even though I wanted us to have sexual intercourse, but I will tell her to wait. PrEP treatment became helpful, I took once a day, I am free so if we desire each other, we can engage without having that concern. Though I am still a little bit concerned but it [is] better now [that] there is PrEP. [I take it] when I come back from work, when I am about to sleep because they [clinical research staff] advised me that if I do not want to see side effects, it is better to take it when I am about to sleep … It has been long since we last used a condom, eish I do not know how to use a condom [but] I think it is important to use both [PrEP and condoms], because it decreases the risks. Because you will never know maybe your system became resistant to the pill, so a condom is also important [but] it feels different … It like washing your hands while wearing gloves, it does do the work but for me it is tight.* – 04 (Partner without HIV)

A partner without HIV who started oral PrEP during the ATI acknowledged using it inconsistently, often referring to online sources that suggested it could be taken around the time of sexual intercourse. While the research team provided oral PrEP to partners without HIV, the participant expressed reluctance to take medication regularly. The partner’s concerns about HIV transmission and PrEP’s effectiveness led to reduced sexual activity and tensions within the relationship. These anxieties led the partner without HIV to prioritize other aspects of life, such as being a good parent.

*Online, I learned that you can take PrEP as needed. As I mentioned, I don’t like taking pills, medication, or supplements – I prefer natural remedies, like traditional medicine. But I did some research on Google because I didn’t want to take PrEP every day. It said you could take PrEP within 24 hours after having sex, as long as it's not more than 24 hours … I took it every day for maybe one or two weeks, but in between, we didn’t have sex every day maybe once or once in a blue moon. I realized I was taking PrEP for free, but I also had concerns about my health, so I did some research to make sure. Okay, I paused for about a week, and then on the day we had sex, I started taking it again, maybe for 4 days, and so on … I knew that if I took PrEP and used protection, I’d be okay. I wasn’t 100% sure, though, which is why I’m saying that sex wasn’t something we did often. My concerns made me not want to have sex as frequently. I would only think about it [sex] a couple of times, and I was sceptical [about PrEP]. I think that’s part of the reason why we fought.* – 04 (Partner without HIV)

Partners with HIV actively sought to better understand their HIV treatment options and the importance of staying undetectable while their partners participated in the ATI. One partner with HIV worried that his taking ART while his partner was on ATI might be difficult for her and highlighted the need for shared responsibility in managing health and reducing risks, especially when discussing condom use during the ATI. Across couples, ongoing communication was central to adjusting behaviors and managing risk during the ATI.

*I am the only one taking the treatment at home now. As my fiancé is taking [the experimental] drugs, so it [is] now up to me when I go to the clinic and ask them to test me and see how my status is. Because I once heard from [Nurse] that if you adhere to treatment; okay it’s fine this virus is able to hide… What we call undetectable… She will bring condoms and tell me that we must use it, the problem is that condoms are small, I even told her. Yes, she did tell me about the [clinical] study, she told me she will pause her treatment for some time. I then asked her; how will she adjust [manage the virus] after pausing her treatment. As I will be continuing with my treatment, would it not affect her as she will be on pause. She said it will not affect her, but we must condomize. So, we used it [condom] but sometimes we did not.* – 07 (Partner with HIV)

To effectively implement partner protections in future trials, partners suggested items such as increased education on various HIV prevention options, ongoing reassurance about PrEP effectiveness, websites with links to accessible information, WhatsApp links, and more opportunities to openly discuss protection options.

*Eh, it does happen that our partners are afraid to tell us their situation but what I have realized, it takes to sit down with your partner and talk to them, if you love them. Same goes to the girlfriends if they want to talk to their partners, they just need to sit them down and talk, nothing beats that. If you can sleep together surely it would not be that hard to sit down together and talk; that there is this and that and fix it. The man will come up with the solution that, no it’s fine, just like me, my partner told me that she joined FRESH, now she has moved to [the clinical trial], and [the clinical trial] has given her a[nother] pill that will fight the virus as she is not taking her treatment anymore. So I understood and congratulated her, I told her I envy [her] to be in the same position. Just that I do not know whether one day there will be a program for men also. Because if this study succeeds surely it can succeed for men also*. – 05 (Partner with HIV)

Partners also suggested that formal workshops and support groups could foster open dialogue around health responsibilities and risk management.

### Social support needs

We explored the roles of partners and family members during the clinical trial, focusing on their essential contributions in providing emotional and practical support, which they viewed as a duty. Partners particularly emphasized their shared commitment and responsibility for the health of their partners enrolled in the ATI trial.

*Okay, I will say I have played an important role since she started, by accepting this trial because for someone else maybe they would have not agreed. I said let us give this way a try, since we started it let us commit ourselves. So that is what we are doing, ever since we started, we will continue until she feels it is dangerous*. – 03 (Partner without HIV)

Some partners recounted their experiences of discovering their loved one’s HIV status and consciously choosing to stay in the relationship while supporting them in their health journey. Partners recounted a process of discovery and acceptance regarding HIV cure research participation, leading to emotional growth and strengthened relationships despite HIV-related stigma. Open communication and mutual respect were viewed as crucial for navigating the complexities of HIV and the clinical trial experience.

Family members, including mothers and siblings, played important roles in providing care and support, helping trial participants rest when needed and encouraging healthy behaviors.

*So, in that case I’d say, when she was on [ART] pause, I told her to exercise so that her body will be active, she must not say I am no longer sick I will drink alcohol and start dating and do bad things. I preferred her to hit the gym, eat healthy, and drink a lot of water so that her body will be alright*. – 10 (Mother)

### Additional recommendations

Partners and family members suggested several ways to increase support for ATI trial participants, including home visits, comprehensive support services, social and educational activities to promote inclusiveness and greater involvement in the clinical research process for loved ones. One male partner mentioned that the ATI trial should not just enroll women but should include men as well.

*Yes, maybe they [study staff] should support them [trial participants] by checking up on them even at their homes. Because you will find there is a time where they face challenges, back at their homes, like if they miss their appointments, go and check them back home if there is something wrong*. – 08 (Partner without HIV)

*Well firstly I can say this programme, it has just started, not so many people know about it. I only knew about it from her and other people who know it, are only those who come here. It would be better if it [the trial] is not for women only, if it can be open to everyone. I am sure many people would wish to be like her, because I am sure this has helped her a lot as much as it has helped me. Can you imagine if it [the trial] was for everyone?* – 03 (Partner without HIV)

The support partners and family members provided was framed as a shared journey toward health and understanding, reinforcing a collective effort toward acceptance, emotional bonds, and well-being. Narratives reflected a complex interplay of love, commitment, challenges, and growth within the relationships of ATI trial participants and their loved ones.

## Discussion

This socio-behavioral study showed that conducting an ATI-inclusive HIV cure trial affected not only the trial participants but also partners and family members of trial participants. Assessment of the impacts of ATIs on partners and family members has provided valuable insights, examined through a relational frame of reference. We found that going through an ATI facilitated emotional bonding among loved ones, mutual adaptation, resilience and support in navigating trial-related challenges. A strong sense of responsibility for the health of trial participants emerged, particularly during the ATI phase. Perceived benefits of trial participation included emotional growth. However, partners and family members also faced trial-related challenges including communication, emotional strain during the ATIs, and HIV-related stigma. Insights regarding partner protections during ATIs came from partners, both with and without HIV.

This is a rare account from sub-Saharan Africa of partners and family members in an ATI-inclusive HIV cure trial, directly responding to calls for inclusion of African populations in cure research (18, 57) and for systematic socio-behavioral documentation alongside biomedical studies (21, 58–60). In comparison, a prior study with 11 ATI trial participants in the U.S. explored the stress, coping strategies, and psychosocial well-being of PWH before, during, and after an ATI, revealing an evolving interplay between individual and environmental factors that influenced participants’ psychosocial outcomes (61). Another study emphasized the importance of building supportive, trusting relationships with clinical research teams and identifying emotional and informational support as stress-buffering mechanisms (61). Despite these insights, far less attention has been devoted to the crucial role of partners and family members in HIV cure research, where mutual coping and resilience are also likely at play.

We found that partners and family members of ATI trial participants largely focused on how the trial affected relationship dynamics, including improved communication, shared emotional support, and strengthened bonds. While they understood the general purpose of the trial, some expressed uncertainties about the specifics of the ATI process. This highlights the need for clear information regarding the trial and the safety and well-being of participants. Additionally, the study revealed the significant protective role that loved ones play in easing participation challenges.

Because partners and family members were not enrolled in ATI trials, sites face limits on direct counseling and service provision. Many sites cannot dispense PrEP to non-participants or assume related liability, which shifts information transfer to the participant and may require referrals that reduce uptake. Practical steps to support loved ones include concise printed or online materials in local languages, brief scripts that participants can use when discussing ATIs, and clear referral pathways for prevention and counseling where available. These actions can address information gaps, build trust, and support participant safety while respecting consent and confidentiality boundaries.

Loved ones of ATI trial participants expressed a complex blend of optimism and caution regarding the perceived risks and benefits of participation, including concerns about relationship risks, emotional burdens, and communication challenges—issues often overlooked in informed consent forms. Our findings align with those of Moodley et al. (62), who noted that HIV cure research in South Africa was perceived as biologically and socially risky. However, in contrast to their study, our research found that loved ones generally showed greater support for trial participation and expressed hope about the possibility of ART-free durable control as a future scientific goal. These findings emphasize the need to proactively address both the possible risks and benefits of trial participation, as well as the potential relational and emotional impacts on both participants and their loved ones. Research teams should carefully consider these factors when designing HIV cure trials to ensure that the emotional, relational, and psychosocial aspects are properly understood and supported. Our study highlights the importance of including socio-behavioral research in ATI-inclusive HIV cure trials that include assessment of the experiences of partners and family members to better understand the extent of impact of ATIs, mitigate harms and provide trial participants with optimal support.

Partners and family members also expressed worries about the health risks, particularly viral rebound, and the emotional burden of supporting their loved ones through the research process. While ongoing clinical monitoring offered some reassurance, the ATI-related concerns noted are aligned with previous focus groups conducted among community members in Durban (22). However, once again, our study highlights the relational and emotional impacts of ATIs, a topic that has received much less attention. Research teams should clearly communicate the safety protocols and scientific rationale for ATIs to participants and incorporate means of ensuring their loved ones have access to the same in a simplified way. Sharing case studies of prior participants who have successfully navigated ATIs could also help reduce anxiety and provide reassurance to loved ones.

Our findings highlight the practical implications of partner protections during ATI trials. For partners without HIV, providing clear, accessible information on effective HIV prevention methods such as PrEP and condom use is essential, as emphasized in prior literature (22, 31–34, 63). While some partners without HIV were already aware of PrEP, others became informed through our study and revealed varying levels of readiness to adopt PrEP as well as inconsistent PrEP use during ATIs. These findings concord with prior research from South Africa, which identified a lack of PrEP knowledge as a significant barrier to its uptake and use (64, 65). To address these gaps, research teams should offer tailored education and proactive support, helping partners of ATI trial participants understand how to effectively use HIV prevention methods during ATIs, while considering local contexts and available resources (22). This could include offering clear guidance on where to access PrEP (66), when it should be taken (67), and under what circumstances it can be safely discontinued. As future trials increase ATI duration, PrEP maintenance for partners will become critically important, with access to long-acting PrEP made available where possible (34, 68). Our study further emphasized the importance of understanding partner protections from the perspectives of both ATI trial participants and partners, using local language and addressing their specific concerns. For example, the research team learned of a dispute between a participant and her partner over PrEP discontinuation, which would be classified as a social harm event (69). This highlights the importance of collecting the social impacts of trials and ATIs to ensure comprehensive support and protection for all involved. Further research is needed on the uptake and use of PrEP among the sex partners of ATI trial participants to better understand the barriers, facilitators, and opportunities for evidence-based intervention.

Partners with HIV voiced concerns about re-transmission risk and their own health during the ATI. These concerns should be anticipated and addressed through information, counseling, and linkage to care. Like partners without HIV, they faced communication and behavior adjustments that influenced relationship dynamics. Proactive attention to both partner groups may improve the feasibility of complex ATI-inclusive trials.

Our study confirmed the presence of mutual coping during ATI-inclusive trials. Previous research has shown that partners in committed relationships view their health as a shared responsibility, often using communal coping strategies to navigate HIV-related challenges (39, 40, 42, 70). Partners in our study similarly expressed concerns about each other’s health and trial-related risks, particularly during the ATI. This highlights the need for dyadic approaches that recognize the interconnectedness of partners’ health decisions. Protocols should include resources designed to address the needs of both trial participants and their partners, fostering a cooperative approach to health management in clinical trials. This will ensure that protocols leverage relational dynamics that influence decision-making at both individual and couple levels (71). Similarly, this approach was described by bioethicist Dawson who advocated for a relational ethics approach to partner protections, arguing that research teams should take on some responsibility for mitigating risks to both ATI trial participants and their partners (41).

To ensure effective support during ATI trials, it is essential to address both the practical and psychosocial needs of participants, recognizing the impact on their partners and families. Although not enrolled in the trial, partners and families of participants are still affected by the trial process, making it critical to define what responsibilities investigators have for protection of affected parties. Our findings reveal a clear need for ongoing emotional and psychological support for affected partners and family members. Provision of this extended support could help trial participants navigate trial processes, foster mutual understanding, and encourage shared responsibility for health. Creating environments that facilitate the involvement of partners and family members, along with tailored interventions and informed by socio-behavioral research sciences, may directly contribute toward successful implementation of complex ATI-inclusive trials. Importantly, our study does not suggest that partners and family members should be automatically involved in ATI trials, as this may not be practical due to financial constraints and participant privacy concerns. Instead, we highlight the importance of using our findings to enhance support for participants, such as personalized counseling and other resources, while emphasizing the need to consider sociocultural contexts when designing ATI trials rather than adopting a one-size-fits-all approach.

Finally, our study highlighted challenges in recruiting partners and family members to participate in HIV cure socio-behavioral research, in a setting where trial participants have not disclosed their HIV status and trial participation due to HIV-related stigma and serve as the gatekeepers for contact. Lessons from our recruitment and engagement with partners and families (Table 3) included the need to accommodate work schedules, meeting participants where they were, and addressing the understanding that the FRESH research site only caters for women. A flexible approach that respected availability, confidentiality, and privacy was required. The background of low HIV disclosure rates in South Africa (72) contributed toward the small samples size in our study. Further studies are needed to better understand the barriers and facilitators to HIV/ATI disclosure. Of note, non-disclosure and serodifferent HIV status between a trial participants and their partners HIV led to an inability of the study team to provide counseling, underscoring the need for evidence-based interventions to support disclosure of HIV status and ATI participation.

**Table 3:** Lessons learned in recruiting and engaging partners and family members (Durban, South Africa, 2022–2024).

*General lessons learned*: Recruitment and engagement efforts must be tailored to the specific needs and circumstances of partners and family members, particularly regarding timing and location of study visits. It is important to meet people where they are (e.g., closer to their home or their work). Understanding participants’ work schedules was crucial. Daytime recruitment can be challenge. We recommend flexibility with study visits while taking personal safety into consideration (e.g., evenings, or weekends). Another strategy could be to write a doctor’s certificate so partners and family members can be excused from work to participate in research. Addressing HIV-associated stigma is essential, as some partners and family members were worried that being seen at the FRESH site would mean they had HIV. Emphasizing confidentiality and privacy was essential, as several partners and family members were initially hesitant to speak about HIV. Some of the proposed options can include that future interviews should be conducted through virtual platforms.
*Lessons learned with partner recruitment*: Male partners often viewed the FRESH research site as being focused primarily on women, leading to their reluctance in participating in a socio-behavioral research interview. Engaging male partners outside of the research site yielded better results. We recommend clear expectations about the nature of the (socio-behavioral) research to avoid creating false hopes. Some male partners with HIV wanted to be included in the HIV cure clinical trial.
*Lessons learned with family members’ recruitment*: We observed FRESH trial participants were hesitant to disclose their HIV status and HIV clinical trial participation to family members, leading to slower recruitment of family members. Female HIV cure trial participants were more comfortable referring mothers and sisters into the socio-behavioral study, likely due to gender dynamics.

**Figure 1:**
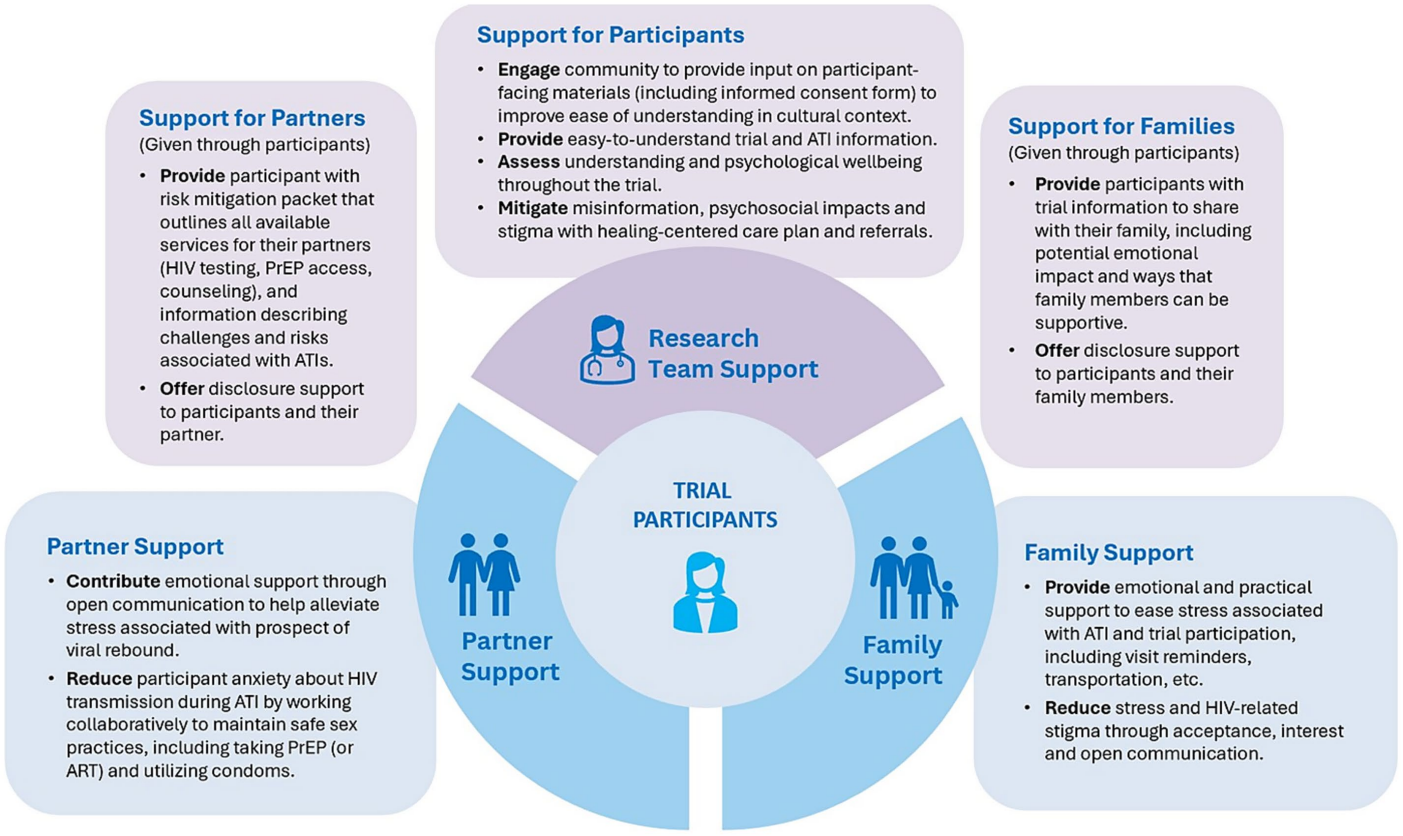
Relational dynamics among research team staff, partners and family members during ATI trial.

Figure 1 illustrates the relational dynamics among research team staff, partners and family members in supporting participants of an HIV cure trial involving an ATI, highlighting the shared investment and responsibility of individuals not formally involved in the trial. Table 4 presents considerations from partners and family members’ perspectives on HIV cure research with ATIs, offering insights that could inform the design and implementation of future ATI trials.

**Table 4:** Summary of considerations emerging from partners’ and family members’ perspectives on HIV cure research with ATIs.

*Awareness of HIV cure clinical trial* *Provide clear and accessible information*: to prevent misconceptions and manage expectations, ensure that trial participants and, where possible, their partners and family members, have access to clear, straightforward information about the HIV cure trial. This should include details about the trial’s purpose, its experimental nature, the procedures involved, potential risks, and possible outcomes. *Acknowledge relational and emotional impacts*: research teams should recognize and address the emotional and relational impacts of participating in the HIV cure trial. This includes understanding both the positive effects (such as improved communication or emotional growth) and the potential negative consequences (such as stress or concerns about health) that could arise for participants and their loved ones. *Highlight the scientific and societal contributions*: emphasize the significant role that HIV cure trial participants play in advancing scientific knowledge and improving HIV control options. Research teams should make it clear that their involvement is not only a personal journey but also a contribution to the broader effort to improve HIV control for future generations, underscoring the societal value of participation.
*Understanding and perceptions of ATIs* *Explain the need for ATIs in research*: research teams should clearly communicate the purpose of ATIs within the context of HIV cure research. This includes explaining why pausing ART is essential for testing experimental interventions and evaluating their effectiveness in controlling HIV without ongoing ART. *Provide information on health risks and safety measures*: clear information should be given about the potential health risks of ATIs, especially the risk of viral rebound. It is important to explain the safety protocols in place to protect participants, such as regular clinical monitoring and criteria for resuming ART if needed, to ensure participants’ health is closely monitored throughout the trial. *Support participants in addressing HIV-related stigma and misconceptions*: research teams should offer strategies for participants, and where possible, their partners and family members, to explain ATI-related processes to others. This will help them address potential HIV-related stigma and misconceptions about the trial within the community, ensuring they have the tools to provide accurate and reassuring information. *Provide case studies to reduce anxiety*: research teams may consider sharing case studies of previous participants who successfully completed ATI-inclusive HIV cure trials. This can help reduce anxiety among current participants and their loved ones, showing real-world examples of how others have navigated the process successfully.
*Partner protections* *Provide clear information on HIV prevention for partners without HIV*: for disclosed partners without HIV, research teams should offer clear and accessible information about HIV prevention methods, such as PrEP and condoms. Additionally, they should provide active support to help partners understand how to use these prevention strategies effectively and maintain their use throughout the trial. Research teams should strive to understand partner protections from the perspectives of those implicated. *Address concerns about PrEP effectiveness and discontinuation*: research teams should address concerns from partners without HIV regarding the effectiveness of PrEP, particularly around its ability to prevent HIV transmission during ATIs. They should also provide guidance on when PrEP can be safely discontinued, if applicable, and under what circumstances. As future ATI trials increase in duration, introducing long-acting PrEP for partners will be necessary. *Acknowledge health-related concerns of partners with HIV*: research teams should recognize that partners with HIV may also have valid concerns regarding their own health, particularly the potential risk of HIV transmission or re-acquisition during ATIs. These concerns should be addressed openly, providing reassurance and appropriate guidance. *Promote open communication to alleviate concerns*: emphasize the importance of open and honest communication between participants, their partners, and research teams. This can help alleviate fears regarding HIV transmission or acquisition during ATIs, ensuring that concerns are addressed promptly and effectively. Future trials should collect systematic information on social impacts of HIV cure trials (69). *Encourage shared responsibility for health management*: research teams should encourage both participants and their partners to take shared responsibility for health management during ATI trials. This includes actively participating in discussions about prevention strategies, treatment adherence, and maintaining overall health during the trial, fostering a collaborative approach to trial participation.
*Social support* *Recognize the critical role of partners and family members*: Partners and family members are essential in providing emotional and practical support to participants throughout HIV cure trials. Their involvement helps ensure that participants feel supported, understood, and cared for during the trial process. *Provide support systems for psychosocial well-being*: research teams should implement support systems, such as counseling services, home visits, regular check-ins, and reminders, to help manage the psychosocial impacts of trial participation. These services should be easily accessible to participants and their families to provide emotional and psychological support. *Address HIV-related stigma and trial misconceptions*: research teams should help participants and their loved ones manage HIV-related stigma by offering information and resources to clarify the trial’s purpose. This includes explaining how the ATI is not HIV treatment default, helping to reduce fears or misconceptions that may arise in the community. *Offer structured support and education for participants and loved ones*: research sites could consider providing more structured support, such as workshops, support groups, or educational sessions. These efforts would help participants and their families develop a mutual understanding of the trial, encourage shared responsibility for health, and foster a sense of community and collaboration in advancing HIV cure research.

### Limitations

We acknowledge several study limitations. One is the small sample size which was due to low HIV disclosure rates as noted above, resulting in only 9 out of 20 cure trial participants referring a partner or family member to the socio-behavioral study (one participant referred both a partner and a sister). Referred partners were cisgender men in steady, committed relationships, thus we did not capture potential impacts on more casual and short-term relationships, nor same-sex relationships. Referred family members were mothers, sisters and a cousin, possibly reflecting gender dynamics within households. As a result, we suspect sampling bias towards more accepting partners and family members. Additionally, with a small sample size, data saturation may not have been achieved (73). On the important topic of partner protections and PrEP usage, we did not ask partners about PrEP access and whether provision of PrEP through the trial site versus referral to their local clinic may have affected usage. In South Africa, it is known that men often delay engaging in healthcare, and that men without HIV or unknown HIV status are even less likely to go to their local government clinics where PrEP can be accessed (74, 75). Since this was a qualitative study, we prioritized depth over generalizability and did not quantify responses to open-ended questions. Translation from *Isi*Zulu to English may have altered some meanings, though two independent translators (N. L., A. Z.) reviewed each transcript for accuracy. Conducting the study at a single site limits generalizability, but we aimed to maintain fidelity to the data in our reporting. The findings are exploratory, and similar research should be conducted with larger, more diverse samples. Future socio-behavioral studies should explore other types of partnerships (e.g., non-steady, non-monogamous, same sex), family relationships, and cultural contexts.

## Conclusion

This study offers rare and critical insights into how partners and family members were affected by an ATI-inclusive HIV cure trial in Durban, South Africa. Our findings show that partners and families are not passive bystanders but are affected by the trial process and actively engaged in provision of support. As one of the first studies to assess the close social network of trial participants, our work provides a glimpse into the extended emotional, social, and practical impacts of ATI-inclusive HIV cure trials and suggests opportunities for risk mitigation and leveraging of an underutilized existing support system. Considerations for partners and family members may contribute toward ATI-inclusive trials that are ethically sound, widely acceptable and responsive to the wider impact on all stakeholders. Through integration of socio-behavioral assessments within ATI-inclusive HIV cure trials, accompanied by effective dissemination of and responsiveness to findings, the HIV cure field can responsibly address critical gaps and challenges associated with ATI implementation. The collective capacity of HIV cure investigators and social scientists to consider partners and family members toward a common good of optimizing participant safety and wellbeing during ATI-inclusive trials stands to advance HIV cure research implementation and outcomes.

## Data Availability

The original contributions presented in the study are included in the article/Supplementary material, further inquiries can be directed to the corresponding author.

## Glossary

ART: antiretroviral treatment
ARV: antiretroviral
ATI: analytical treatment interruption
bNAbs: broadly neutralizing antibodies
BREC: Biomedical Research Ethics Committee
CBPR: community-based participatory research
COREQ: Consolidated Criteria for Reporting Qualitative Research
FRESH: Females Rising through Education, Support and Health
GCP: Good Clinical Practice
HIV: human immunodeficiency virus
IDI: in-depth interview
IPV: intimate partner violence
IRB: Institutional Review Board
MGB: Mass General Brigham
PrEP: pre-exposure prophylaxis
PWH: people with HIV
RNA: ribonucleic acid
TLR: toll-like receptor
UCSD: University of California San Diego
UKZN: University of Kwa-Zulu Natal

## References

[1] UNAIDS. Fact sheet 2024: global HIV statistics. (2024). Available online at: https://www.unaids.org/sites/default/files/media_asset/UNAIDS_FactSheet_en.pdf (accessed September 20, 2024)

[2] Christopher P Murray JL. Global, regional, and national incidence, prevalence, and mortality of HIV, 1980–2017, and forecasts to 2030, for 195 countries and territories: a systematic analysis for the global burden of diseases, injuries, and risk factors study 2017. Lancet HIV. (2019) 6:e831–59. doi: 10.1016/S2352-3018(19)30196-1 31439534 PMC6934077

[3] HSRC Press. South African national HIV prevalence, incidence, behaviour and communications survey, 2017. 2019. Available online at: https://repository.hsrc.ac.za/bitstream/20.500.11910/15052/1/11091.pdf (accessed September 6, 2024)

[4] Karim QA Kharsany ABM Frohlich JA Werner L Mlotshwa M Madlala BT . HIV incidence in young girls in KwaZulu-Natal, South Africa. Public health imperative for their inclusion in HIV biomedical intervention trials. AIDS Behav. (2012) 16:1870–6. doi: 10.1007/s10461-012-0209-y, 22618892 PMC3460144

[5] Kharsany ABM Cawood C Lewis L Yende-Zuma N Khanyile D Puren A . Trends in HIV prevention, treatment, and incidence in a hyperendemic area of KwaZulu-Natal, South Africa. JAMA Netw Open. (2019) 2:e1914378. doi: 10.1001/jamanetworkopen.2019.14378 31675082 PMC6826647

[6] Abdool Karim Q Havlir D Phanuphak N. Putting women in the Centre of the Global HIV response is key to achieving epidemic control! J Int AIDS Soc. (2020) 23:23–5. doi: 10.1002/jia2.25473, 32142213 PMC7059773

[7] Mannell J Willan S Shahmanesh M Seeley J Sherr L Gibbs A. Why interventions to prevent intimate partner violence and HIV have failed young women in Southern Africa. J Int AIDS Soc. (2019) 22:1–6. doi: 10.1002/jia2.25380, 31441229 PMC6706780

[8] Mthembu J Mabaso M Reis S Zuma K Zungu N. Prevalence and factors associated with intimate partner violence among the adolescent girls and young women in South Africa: findings the 2017 population-based cross-sectional survey. BMC Public Health. (2021) 21:1–9. doi: 10.1186/s12889-021-11183-z, 34134666 PMC8210348

[9] Gibbs A Dunkle K Willan S Jama-Shai N Washington L Jewkes R. Are women’s experiences of emotional and economic intimate partner violence associated with HIV-risk behaviour? A cross-sectional analysis of young women in informal settlements in South Africa. AIDS Care. (2019) 31:667. doi: 10.1080/09540121.2018.1533230, 30409025

[10] Hlongwa M Peltzer K Hlongwana K. Risky sexual behaviours among women of reproductive age in a high HIV burdened township in KwaZulu-Natal, South Africa. BMC Infect Dis. (2020) 20:563. doi: 10.1186/s12879-020-05302-1 32738895 PMC7395408

[11] Muula A. HIV infection and AIDS among young women in South Africa. Croat Med J. (2008) 49:423–35. doi: 10.3325/cmj.2008.3.423, 18581623 PMC2443629

[12] Palanee-Phillips T Rees HV Heller KB Ahmed K Batting J Beesham I . High HIV incidence among young women in South Africa: data from a large prospective study. PLoS One. (2022) 17:e0269317. doi: 10.1371/journal.pone.0269317 35657948 PMC9165791

[13] Wingood GM Reddy P Peterson SH DiClemente RJ Nogoduka C Braxton N . HIV stigma and mental health status among women living with HIV in the Western cape, South Africa. S Afr J Sci. (2008) 104:237–40.

[14] Psaros C Milford C Smit JA Greener L Mosery N Matthews LT . HIV prevention among young women in South Africa: understanding multiple layers of risk. Arch Sex Behav. (2018) 47:1969–82. doi: 10.1007/s10508-017-1056-8, 29134422 PMC5966340

[15] Kagee A Remien R Berkman A Hoffman S Campos L Swartz L. Structural barriers to ART adherence in southern Africa: challenges and potential ways forward. Glob Public Health. (2011) 6:83–97. doi: 10.1080/17441691003796387 20509066 PMC3056922

[16] Dybul M Attoye T Baptiste S Cherutich P Dabis F Deeks S . The case for an HIV cure and how to get there. Lancet HIV. (2021) 8:e51–8. doi: 10.1016/S2352-3018(20)30232-0, 33271124 PMC7773626

[17] Lewin SR Attoye T Bansbach C Doehle B Dubé K Dybul M . Multi-stakeholder consensus on a target product profile for an HIV cure. Lancet HIV. (2021) 8:e42–50. doi: 10.1016/S2352-3018(20)30234-4, 33271125 PMC7773628

[18] Ndung’u T McCune JM Deeks SG. Why and where an HIV cure is needed and how it might be achieved. Nature. (2019) 576:397–405. doi: 10.1038/s41586-019-1841-8, 31853080 PMC8052635

[19] Ndung’u T Dong KL Kwon DS Walker BD. A FRESH approach: combining basic science and social good. Sci Immunol. (2018) 3:eaau2798. doi: 10.1126/sciimmunol.aau2798, 30217812 PMC7593829

[20] Dong K Moodley A Kwon D Ghebremichael M Dong M Ismail N . Detection and treatment of Fiebig 1 HIV-1 infection in young at-risk women in South Africa: a prospective cohort study. Lancet HIV. (2018) 5:e35–44. doi: 10.1016/S2352-3018(17)30146-7 28978417 PMC6506720

[21] Miall A McLellan R Dong K Ndung’u T Saberi P Sauceda JA . Bringing social context into global biomedical HIV cure-related research: an urgent call to action. J Virus Erad. (2021) 8:100062. doi: 10.1016/j.jve.2021.100062 35169489 PMC8829132

[22] Dubé K Mthimkhulu D Ngcobo W Mindry D Maphalala L Pillay V . “With this study, we have hope that something is coming”: community members’ perceptions of HIV cure-related research in Durban, South Africa—a qualitative focus group study. HIV Res Clin Pract. (2023) 24:1–13. doi: 10.1080/25787489.2023.2243046 PMC10433450 37555592

[23] Sandel DA Rutishauser RL Peluso MJ. Post-intervention control in HIV immunotherapy trials. Curr Opin HIV AIDS. (2024) 19:1–10. doi: 10.1097/COH.0000000000000890 39494630 PMC11620322

[24] SenGupta D Brinson C DeJesus E Mills A Shalit P Guo S . The TLR7 agonist vesatolimod induced a modest delay in viral rebound in HIV controllers after cessation of antiretroviral therapy. Sci Transl Med. (2021) 13:1–16. doi: 10.1126/scitranslmed.abg3071, 34162752

[25] Julg B Dee L Ananworanich J Barouch D Bar K Caskey M . Recommendations for analytical treatment interruptions in HIV research trials. Report of a consensus meeting. Lancet HIV. (2019) 6:e259–68. doi: 10.1016/S2352-3018(19)30052-9 30885693 PMC6688772

[26] Clarridge KE Blazkova J Einkauf K Petrone M Refsland W Justement JS . Effect of analytical treatment interruption and reinitiation of antiretroviral therapy on HIV reservoirs and immunologic parameters in infected individuals. PLoS Pathog. (2018) 14:e1006792. doi: 10.1371/journal.ppat.1006792, 29324842 PMC5764487

[27] Li JZ Aga E Bosch RJ Pilkinton M Kroon E MacLaren L . Time to viral rebound after interruption of modern antiretroviral therapies. Clin Infect Dis. (2022) 74:865–870. doi: 10.1093/cid/ciab54 34117753 PMC8906742

[28] Hurst J Hoffmann M Pace M Williams JP Thornhill J Hamlyn E . Immunological biomarkers predict HIV-1 viral rebound after treatment interruption. Nat Commun. (2015) 6:8495. doi: 10.1038/ncomms9495 26449164 PMC4633715

[29] Pannus P Rutsaert S De Wit S Allard SD Vanham G Cole B . Rapid viral rebound after analytical treatment interruption in patients with very small HIV reservoir and minimal on-going viral transcription. J Int AIDS Soc. (2020) 23:e25453. doi: 10.1002/jia2.25453 32107887 PMC7046528

[30] Lau JSY Cromer D Pinkevych M Lewin SR Rasmussen TA McMahon JH . Balancing statistical power and risk in HIV cure clinical trial design. J Infect Dis. (2022) 226:236–45. doi: 10.1093/infdis/jiac032 35104873 PMC9400422

[31] Peluso MJ Dee L Campbell D Taylor J Hoh R Rutishauser RL . A collaborative, multidisciplinary approach to HIV transmission risk mitigation during analytic treatment interruption. J Virus Erad. (2020) 6:34–7. doi: 10.1016/S2055-6640(20)30009-1, 32175090 PMC7043899

[32] Dubé K Kanazawa JT Dee L Taylor J Campbell DM Brown BJ . Ethical and practical considerations for mitigating risks to sexual partners during analytical treatment interruptions in HIV cure-related research. HIV Res Clin Pract. (2021) 22:14–30. doi: 10.1080/25787489.2021.1902116 33757411 PMC8272285

[33] Dubé K Morton T Fox L Dee L Palm D Villa TJ . A partner protection package for HIV cure-related trials involving analytical treatment interruptions. Lancet Infect Dis. (2023) 23:e418–30. doi: 10.1016/S1473-3099 37295453 PMC10543569

[34] Dubé K Villa TJ Freshwater W Mauk B Rid A Peluso MJ. Partner protections in HIV cure-related trials involving analytical treatment interruption: updated toolkit to mitigate HIV transmission risk. J Virus Erad. (2024) 10:100386–6. doi: 10.1016/j.jve.2024.100386, 39364082 PMC11447310

[35] Dubé K Sylla L Dee L Taylor J Evans D Bruton C . Research on HIV cure: mapping the ethics landscape. PLoS Med. (2017) 14:e1002470. doi: 10.1371/journal.pmed.1002470 29220353 PMC5722280

[36] Lelièvre JD Hocqueloux L. Unintended HIV-1 transmission to a sex partner in a study of a therapeutic vaccine candidate. J Infect Dis. (2019) 220:S5–6. doi: 10.1093/infdis/jiz012 30779842 PMC6603976

[37] Ugarte A Romero Y Tricas A Casado C Garcia F Leal L. Unintended HIV-1 infection during analytical treatment interruption. J Infect Dis. (2020) 221:1740–2. doi: 10.1093/infdis/jiz611, 31742347

[38] Dubé K Ndukwe SO Korolkova A Dee L Sugarman J Sauceda JA. Participant experiences in a combination HIV cure-related trial with extended analytical treatment interruption in San Francisco, United States. HIV Res Clin Pract. (2024) 25:1–15. doi: 10.1080/25787489.2024.2312318, 38348830 PMC10951555

[39] Campbell D Dubé K Cowlings P Dionicio P Tam R Agarwal H . “It comes altogether as one”: perceptions of analytical treatment interruptions and partner protections among racial, ethnic, sex and gender diverse HIV serodifferent couples in the United States. BMC Public Health. (2022) 22:1317. doi: 10.1186/s12889-022-13528-8 35810288 PMC9270765

[40] Dubé K Agarwal H Stockman JK Auerbach JD Sauceda JA Conroy AA . “I would absolutely need to know that my partner is still going to be protected”: perceptions of HIV cure-related research among diverse HIV serodifferent couples in the United States. AIDS Res Hum Retrovir. (2022) 39:400–13. doi: 10.1089/aid.2022.0036 35972752 PMC10387158

[41] Dawson L. Human immunodeficiency virus transmission risk in analytical treatment interruption studies: relational factors and moral responsibility. J Infect Dis. (2019) 220:S12–5. doi: 10.1093/infdis/jiz090, 31264689 PMC6775575

[42] Conroy A Gamarel K Neilands T Dilworth S Darbes L Johnson M. Relationship dynamics and partner beliefs about viral suppression: a longitudinal study of male couples living with HIV/AIDS (the duo project). AIDS Behav. (2016) 20:1572–83. doi: 10.1007/s10461-016-1423-9, 27150895 PMC4920065

[43] Dubé K Patel H Concha-Garcia S Perry K Mathur K Javadi S . Perceptions of next-of-kin/loved ones about last gift rapid research autopsy study enrolling people with HIV/AIDS at the end-of-life: a qualitative interview study. AIDS Res Hum Retrovir. (2020) 36:1033–46. doi: 10.1089/aid.2020.0025, 32449624 PMC7703245

[44] Javadi SS Mathur K Concha-Garcia S Patel H Perry K Lo M . Attitudes and perceptions of next-of-kin/loved ones toward end-of-life HIV cure-related research: a qualitative focus group study in Southern California. PLoS One. (2021) 16:e0250882. doi: 10.1371/journal.pone.0250882, 33961653 PMC8104928

[45] Power J Dowsett GW Westle A Tucker JD Hill S Sugarman J . The significance and expectations of HIV cure research among people living with HIV in Australia. PLoS One. (2020) 15:e0229733. doi: 10.1371/journal.pone.0229733, 32130262 PMC7055878

[46] Noorman MAJ de Wit JBF Marcos TA Stutterheim SE Jonas KJ den Daas C. Engagement of HIV-negative MSM and partners of people with HIV in HIV cure (research): exploring the influence of perceived severity, susceptibility, benefits, and concerns. AIDS Care. (2024) 36:211–22. doi: 10.1080/09540121.2024.2307381, 38319908

[47] Maman S Medley A. Gender dimensions of HIV status disclosure to sexual partners: rates barriers and outcomes gender dimensions of HIV status disclosure to sexual partners: rates, barriers and outcomes. Reprod Health Matters. (2006) 14:234.

[48] Dubé K Perez-Brumer A. Call for justice-informed HIV cure trials with ATIs. Lancet HIV. (2024) 11:e137–9. doi: 10.1016/S2352-3018(24)00002-X 38281500 PMC10922922

[49] Wallterstein N Duran B Oetzel J Minkler M. Community-based participatory research for health: advancing social and health equity. San Francisco, CA: Jossey-Bas (2017).

[50] Cait C. Relational theory In: Coady N Lehmann P, editors. Theoretical perspectives for direct social work practice: a generalist-eclectic approach. 3rd ed. New York, NY, USA: Springer Publishing Company (2016). 185–202.

[51] Care RM. Family policy and social citizenship in South Africa. J Comp Fam Stud. (2017) 48:327–38. doi: 10.3138/jcfs.48.3.327, 40673054

[52] Hall K Richter L Mokomane Z Lake L Children, families and the state. Collaboration and contestation Cape Town Children’s Institute, University of Cape Town 2018 1–172

[53] Duby Z Bergh K Jonas K Reddy T Bunce B Fowler C . “Men rule… this is the Normal thing. We normalise it and it’s wrong”: gendered power in decision-making around sex and condom use in heterosexual relationships amongst adolescents and young people in South Africa. AIDS Behav. (2023) 27:2015–29. doi: 10.1007/s10461-022-03935-8, 36441410 PMC10149448

[54] Creswell J. Research design. Qualitative, quantitative and mixed methods approaches. Thousand Oaks, CA: SAGE Publications (2014). 273 p.

[55] Hsieh HF Shannon SE. Three approaches to qualitative content analysis. Qual Health Res. (2005) 15:1277–88. doi: 10.1177/1049732305276687, 16204405

[56] Corbin J Strauss A. Grounded theory research: procedures, canons, and evaluative criteria. Qual Sociol. (1990) 13:3–21. doi: 10.1007/BF00988593

[57] Bonney EY Lamptey H Kyei GB. HIV cure: an acceptability scientific agenda. Curr Opin HIV AIDS. (2023) 18:12–7. doi: 10.1097/COH.0000000000000771, 36503877 PMC9757853

[58] Grossman CI Ross AL Auerbach JD Ananworanich J Dubé K Tucker JD . Towards multidisciplinary HIV-cure research: integrating social science with biomedical research. Trends Microbiol. (2016) 24:5–11. doi: 10.1016/j.tim.2015.10.011, 26642901 PMC4698010

[59] Dubé K Auerbach JD Stirratt MJ Gaist P. Applying the behavioural and social sciences research (BSSR) functional framework to HIV cure research. J Int AIDS Soc. (2019) 22:e25404. doi: 10.1002/jia2.25404, 31665568 PMC6820877

[60] Noorman MAJ de Wit JBF Marcos TA Stutterheim SE Jonas KJ den Daas C. The importance of social engagement in the development of an HIV cure: a systematic review of stakeholder perspectives. AIDS Behav. (2023) 27:3789–3812. doi: 10.1007/s10461-023-04095-z 37329470 PMC10589186

[61] Hill M Garcia LR Nguyen E Korolkova A Cohn L Rodriguez A . Evaluating the psychosocial experiences of participants in HIV cure research before, during, and after analytical treatment interruptions: a longitudinal qualitative study in the United States. Soc Sci Med. (2025) 366:117644. doi: 10.1016/j.socscimed.2024.117644, 39754855 PMC11915557

[62] Moodley K Rossouw T Staunton C Colvin CJ. Synergies, tensions and challenges in HIV prevention, treatment and cure research: exploratory conversations with HIV experts in South Africa. BMC Med Ethics. (2016) 17:26. doi: 10.1186/s12910-016-0109-1 27137204 PMC4853862

[63] Peluso MJ Dee L Shao S Taylor J Campbell D Collins S . Operationalizing HIV cure-related trials with analytic treatment interruptions during the SARS-Cov-2 pandemic: a collaborative approach. Clin Infect Dis. (2021) 72:1843–9. doi: 10.1093/cid/ciaa1260 32841311 PMC7499539

[64] Ntinga X Isehunwa OO Msimango LI Smith PM Matthews LT Van Heerden A. Perceptions of pre-exposure prophylaxis (PrEP) for HIV prevention among men living with HIV in the context of reproductive goals in South Africa: a qualitative study. BMC Public Health. (2024) 24:1–8. doi: 10.1186/s12889-024-18118-4, 38389039 PMC10882859

[65] Shamu S Shamu P Khupakonke S Farirai T Chidarikire T Guloba G . Pre-exposure prophylaxis (PrEP) awareness, attitudes and uptake willingness among young people: gender differences and associated factors in two south African districts. Glob Health Action. (2021) 14:1886455. doi: 10.1080/16549716.2021.1886455 33606603 PMC7899653

[66] Hlongwa M Basera W Nicol E. Comparing PrEP initiation rates by service delivery models among high risk adolescent boys and young men in KwaZulu-Natal, South Africa: findings from a population-based prospective study. BMC Public Health. (2024) 24:1–7. doi: 10.1186/s12889-024-18660-1, 38658900 PMC11043044

[67] Musinguzi N Kidoguchi L Mugo NR Ngure K Katabira E Celum CL . Adherence to recommendations for ART and Targeted PrEP use among HIV serodiscordant couples in East Africa: the “PrEP as a bridge to ART” strategy. BMC Public Health. (2020) 20:1621. doi: 10.1186/s12889-020-09712-3 33115478 PMC7594426

[68] Meyers K Price D Golub S. Behavioral and social science research to support accelerated and equitable implementation of long-acting Preexposure prophylaxis. Curr Opin HIV AIDS. (2020) 15:66–72. doi: 10.1097/COH.0000000000000596, 31644482 PMC6924276

[69] Allen M Lau CY. Social impact of preventive HIV vaccine clinical trial participation: a model of prevention, assessment and intervention. Soc Sci Med. (2008) 66:945–51. doi: 10.1016/j.socscimed.2007.10.019 18162272

[70] Karney BR Hops H Redding CA Reis HT Rothman AJ Simpson JA. A framework for incorporating dyads in models of HIV-prevention. AIDS Behav. (2010) 14:189–203. doi: 10.1007/s10461-010-9802-0, 20838872 PMC4156876

[71] Dubé K Kanazawa JT Campbell C Boone CA Maragh-Bass A Campbell DM . Considerations for increasing racial, ethnic, gender and sexual diversity in HIV cure-related research with analytical treatment interruptions: a qualitative inquiry. AIDS Res Hum Retrovir. (2022) 38:50–63. doi: 10.1089/aid.2021.0023, 33947268 PMC8785755

[72] Bhatia DS Harrison AD Kubeka M Milford C Kaida A Bajunirwe F . The role of relationship dynamics and gender inequalities as barriers to HIV-Serostatus disclosure: qualitative study among women and men living with HIV in Durban, South Africa. Front Public Health. (2017) 5:188. doi: 10.3389/fpubh.2017.00188 28824897 PMC5534462

[73] Guest G Bunce A Johnson L. How many interviews are enough?: an experiment with data saturation and variability. Field Methods. (2006) 18:59–82. doi: 10.1177/1525822X05279903

[74] Randera-Rees S Safari W Gareta D Herbst K Baisley K Grant A. Can we find the missing men in clinics? Clinic attendance by sex and HIV status in rural South Africa. Wellcome Open Res. (2023) 6:169. doi: 10.12688/wellcomeopenres.16702.2 37767058 PMC10521066

[75] Daniels J Medina-Marino A Glockner K Grew E Ngcelwane N Kipp A. Masculinity, resources, and retention in care: South African men’s behaviors and experiences while engaged in TB care and treatment. Soc Sci Med. (2021) 270:113639. doi: 10.1016/j.socscimed.2020.113639, 33493956 PMC8445063

