# Supplementary material for: Impact of an analytical treatment interruption on partners and family members of trial participants in Durban, South Africa: a qualitative study
Source: Front Public Health. 2025 Dec 15;13:1662141. doi: 10.3389/fpubh.2025.1662141 (PMC12745266; doi:10.3389/fpubh.2025.1662141)
Supplement: Supplementary file 1 [file Table_1.docx]

**Supplementary Table 1: Consolidated Criteria for Reporting Qualitative Research (COREQ) Checklist**

| **Item No** | | **Guide Questions/Description** | **Completed** | **Reported on Page #** |  |
| --- | --- | --- | --- | --- | --- |
| **Domain 1: Research team and reflexivity** | | | | |  |
| **Personal Characteristics** | | | | |  |
| 1. Interviewer(s) | | Which author/s conducted the interviews? | Yes | Page 5 |  |
| 2. Credentials | | What were the researcher’s credentials? E.g., PhD, MD | Yes | Page 4 |  |
| 3. Occupation | | What was their occupation at the time of the study? | Yes | Pages 4 – 5 |  |
| 4. Gender | | Was the researcher male or female? | Yes | Page 4 |  |
| 5. Experience and training | | What experience or training did the researcher have? | Yes | Page 4 |  |
| **Relationship with participants** | | | | |  |
| 6. Relationship established | | Was a relationship established prior to study commencement? | Yes | Page 4 |  |
| 7. Participant knowledge of the interviewer | | What did the participants know about the researcher? e.g. personal goals, reasons for doing the research? | Yes | Page 4 |  |
| 8. Interviewer characteristics | | What characteristics were reported about the interviewer? e.g. Bias, assumptions, reasons and interests in the research topic | Yes | Page 4 |  |
| **Domain 2: study design** | | | |  |  |
| **Theoretical framework** | | | |  |  |
| 9. Methodological orientation and Theory | What methodological orientation was stated to underpin the study? e.g. grounded theory, discourse analysis, ethnography, phenomenology, content analysis | Yes | Page 5 |  |  |
| **Participant selection** | | | |  |  |
| 10. Sampling | How were participants selected? e.g., purposive, convenience, consecutive, snowball | Yes | Page 4 |  |  |
| 11. Method of approach | How were participants approached? e.g., face-to-face, telephone, mail, email | Yes | Page 4 |  |  |
| 12. Sample size | How many participants were in the study? | Yes | Page 5 |  |  |
| 13. Non-participation Setting | How many people refused to participate or dropped out? Reasons? | Yes | Page 5 – 6 |  |  |
| 14. Setting of data collection | Where was the data collected? e.g., home, clinic, workplace | Yes | Pages 4 – 5 |  |  |
| 15. Presence of nonparticipants | Was anyone else present besides the participants and researchers? | N/A | N/A |  |  |
| 16. Description of sample | What are the important characteristics of the sample? e.g. demographic data, date | Yes | Page 5  Table 2 |  |  |
| **Data collection** | | | |  | No |
| 17. Interview guide | Were questions, prompts, and guides provided by the authors? Was it pilot tested? | Yes | Page 4  Table 1 |  |  |
| 18. Repeat interviews | Were repeat interviews carried out? If yes, how many? | Yes | N/A |  |  |
| 19. Audio/visual recording | Did the research use audio or visual recording to collect the data? | Yes | Pages 4 – 5 |  |  |
| 20. Field notes | Were field notes made during and/or after the interview? | Yes | Pages 4 – 5 |  |  |
| 21. Duration | What was the duration of the interviews or focus group? | Yes | Page 5 |  |  |
| 22. Data saturation | Was data saturation discussed? | Yes | Page 13 (Limitations) |  |  |
| 23. Transcripts returned | Were transcripts returned to participants for comment and/or correction? | Yes | Page 5 |  |  |
| **Domain 3: analysis and findings** | | | |  |  |
| **Data analysis** | | | |  |  |
| 24. Number of data coders | How many data coders coded the data? | Yes | Page 5 |  |  |
| 25. Description of the coding tree | Did the authors provide a description of the coding tree? | Yes | Page 5 |  |  |
| 26. Derivation of themes | Were themes identified in advance or derived from the data? | Yes | Page 5 |  |  |
| 27. Software | What software, if applicable, was used to manage the data? | Yes | Page 5 |  |  |
| 28. Participant checking | Did participants provide feedback on the findings? | Yes | Page 5 |  |  |
| **Reporting** | | | |  |  |
| 29. Quotations presented | Were participant quotations presented to illustrate the themes/findings? Was each quotation identified? e.g., participant number | Yes | Pages 6 – 11  Supplementary Table 2 |  |  |
| 30. Data and findings consistent | Was there consistency between the data presented and the findings? | Yes | Pages 6 – 11 |  |  |
| 31. Clarity of major themes | Were major themes clearly presented in the findings? | Yes | Pages 6 – 11 |  |  |
| 32. Clarity of minor themes | Is there a description of diverse cases or a discussion of minor themes? | Yes | Pages 6 – 11 |  |  |

**Developed from**:

Tong A, Sainsbury P, Craig J. Consolidated criteria for reporting qualitative research (COREQ): a 32-item checklist for interviews and focus groups. International Journal for Quality in Health Care. 2007. Volume 19, Number 6: pp. 349 – 357

**Supplementary Table 2: Additional or Supplementary Quotes – Perspectives from Partners and Family Members on HIV Cure Research with ATIs (Durban, South Africa)**

| **Themes and** | **Subthemes** | **Participant Number** | **Informant Type** | **Quotations** |  |
| --- | --- | --- | --- | --- | --- |
| **General Experiences of the HIV Cure Trial** | | | | | |
| **Understanding and Impact of Clinical Trial** | | | | | |
| Awareness of trial’s purpose | | **08** | Partner without HIV | *What I understand is that you are trying to find medication that will suppress the virus, that’s all I can say.* |  |
|  |  | **05** | Partner with HIV | *She joined here [FRESH] long time ago… I always supported her. She told me about the study [trial]… Ever since she joined the study, there is nothing she does not tell me when she comes back from her visits. There is a lot I know about this study because even when she went for lymph node procedure, she told me. She told me about the pill and drip also, and how long do they monitor them before they go home… All in all, they want to find cure to this disease.* |  |
|  |  | **07** | Partner with HIV | *I think their purpose is to do something better, I think it’s clear that they are trying to fight the virus.* |  |
|  |  | **06** | Mother | *That means the purpose is to see, if what they are giving her [trial drugs] does work, so that it can be used future to help others. Because if they see that it is not working, it cannot go on to other people.* |  |
|  |  | **01** | Sister | *The aim for this study [trial], is that at the end people end not having to take ARVs [ART].* |  |
|  |  | **02** | Cousin | *The purpose is to find out if they can live as young females without having to take ARVs [ART]… I know that if she is not home she is here, because this started as FRESH and now it is [the clinical trial] … Now I know that there is [an experimental product] and also ATI and some other things I used to see, but I could not always follow her and ask questions… And I also know about the pregnancy tests which are always done.* |  |
| Impact on relationships | Strengthened bonds | **05** | Partner with HIV | *Our relationship is okay, we do not discriminate each other, we are not swearing at one another. Our relationship is okay, we do not discriminate each other, we are not swearing at one another. We do have small fights just like any relationship, sometimes in relationships there are many challenges, but for us, we are still okay.* |  |
| Need for clear information and clear guidance | | **04** | Partner without HIV | *I was skeptical until she came back with pamphlets, and I read it, then I realized that this will help her to live a normal life. She can accept herself and be free, because I have noticed that if we address this topic, I see that she feels uncomfortable.* |  |
|  |  | **08** | Partner without HIV | *What I can say is that I was not familiar with it but when you briefed me, I got an understanding that it is a new method of trying to suppress the virus, this new method can help people not to take treatment day by day.* |  |
|  |  | **06** | Mother | *Yes, she told me, I was scared, I told her, it is said when you have started treatment you must not stop it, or you are going to have a problem. She told me there are other drugs she will be given as a substitute to see if the virus remains suppressed or not. I was afraid, but then because this is led by doctors, I said it fine she can do it. If something happens, you will call them right away, she confirmed that she has the numbers. That they want to see, what will happen to her health if she stops her treatment then she stopped. I did not see any trouble… [then] she told me, ‘Mom, they have told me to take my treatment again’* |  |
| **Motivations and Feelings about Participation** | | | | | |
| Learning about one’s health | | **06** | Mother | *This study has a lot of lessons, if she has not joined FRESH, she would have not known about her health. She went there to learn, and then this programme [Gilead] came, and she joined. She told me about it, and I encouraged her to join, it was very helpful because if it was not for it, maybe she would have been alive today.* |  |
| Desire for self-improvement and empowerment | | **04** | Partner without HIV | *I have never asked her, to tell you the truth, I think she is that person who always wants to improve herself. She used to go to the gym and eat healthy, like fruits. So, I know deep down, she wants to improve her life somehow, that is what we want.* |  |
|  |  | **07** | Partner with HIV | *Uh, she wants to better her life, to see if she can fight this by this new way. Okay I will stop the pills and see if she will still have this thing. Because she did say she would have not qualified to join if it was not suppressed but it was suppressed so she joined the study.* |  |
| **Feelings About Loved One’s Trial Participation** | | | | | |
| Acceptance | | **03** | Partner without HIV | *Okay, since she started, after they have diagnosed her. She told me that there is a program she would like to join, so I said if she feels it [is] the right thing, I cannot say I am not right about it. I must support her. So, she explained to me about the study [trial], where they are trying to suppress the virus to a point, they do not need to take ARVs [ART]. So, I said that is a good opportunity that she must take it****…*** *After some time, I will tell her to take her pills, and she will ask me if I still remember that she is on pause. What can help us is to set alarm if ever it is found that she must take her meds again. We are expecting anything because this is research at the end of the day. It can be successful or unsuccessful, so far everything is normal.* |  |
|  |  | **07** | Partner with HIV | *So, we are both taking treatment, since then we had no problems ‘til today. And then one day she told me, that she has been introduced to some new study [trial], I encouraged her to join, that this could be helpful to her.* |  |
|  |  | **10** | Mother | *I have heard about it [and] I did not have a problem with her participation.* |  |
| Hope and happiness | | **08** | Partner without HIV | *I feel happy that she is taking initiative about her life in fighting the virus... I can see my wife has gained weight now.* |  |
|  |  | **01** | Sister | *I am so happy because after she joined the study, I saw a lot of improvement from her side as a person who was taking ARVs [ART].* |  |
| Perceived benefits | Clinical monitoring | **02** | Cousin | *From what I have heard today is that they come here every week… and they are being checked, so it will be discovered early if anything changes when it can still be avoided.* |  |
|  | Health improvements – weight gain | **05** | Partner with HIV | *So, she joined FRESH, who referred her to [the clinical trial] and all that. So now she is not taking her pill, but ended up taking [experimental] drugs, so this [experimental drug] is able to fight the virus in her because she has not lost weight. What I have noticed also, is that, there are these leggings and jeans of her that do not fit anymore. So, I see she has gained weight.* |  |
|  | Health improvements – weight gain | **08** | Partner without HIV | *What I can say is I feel happy that she is taking initiative about her life in fighting the virus. Off which she is trying in every way to lead a normal life, I can see my wife has gained weight now… she has appetite, the thing is we are not living together so I will never know other benefits. But when it comes to food, she is now having an appetite unlike before.* |  |
|  | Not having to take ART for limited period | **01** | Sister | *What I can say is that since she was taking ARVs [ART] she used to forget them and not use them, but since coming here, as she is attending, there is something that you gave her which helps her, since she is also not taking her ARVs [ART] saying that you have stopped her.* |  |
|  |  | **09** | Sister | *Yes, there is, that as she has started the study, she sees the difference as she is no longer taking pills, she has started to see that it’s working.* |  |
| Worries about the trial | Health concerns | **04** | Partner without HIV | *As I said, I was thinking especially for the [our] child, I do not want my child to grow up without her mother. So, my concern is that, when she is not taking her treatment, would the viral load not increase. Sometimes I feel like this is a risk.* |  |
|  | Communication challenges | **04** | Partner without HIV | *I talk, as I said she is the one who is uncomfortable about talking. So, if I ask her such questions, she does not respond because I know she also does not know. She is also just hoping that this [clinical] trial will help her.* |  |
| No worry | | **09** | Sister | *For now, I see no danger. Everything is going well… There’s nothing.* |  |
| Overall trust in research process | | **09** | Sister | *I felt better because the cure to help people will be found [the trial will help find a cure], and she will also get help in this study.* |  |
| **Informational and Emotional Needs** | | | | | |
| Questions about health risks | | **01** | Sister | *Is there any risk you can have since you stop your ARVs [ART], while trying to use these products?* |  |
| Questions about HIV acquisition risks | | **03** | Partner without HIV | *When it comes to risks, I think if it happens that I get infected [acquire HIV], that could be the possible risk. I have long accepted that, but it is a risk at the end.* |  |
| Emotional responses and needs | Fear and anxiety | **09** | Sister | *It was scary because we did not know that it was going to work or not but it did work.* |  |
| Understanding of the research process | Concerns about community impact | **08** | Partner without HIV | *What I would like to know is as we know there are people from our neighborhood who defaulted from treatment, and they are facing some difficulties. They are sick and become paralyzed, how would you prevent such circumstances from happening.* |  |
| Informational needs regarding potential trial outcomes | | **04** | Partner without HIV | *My question is, is there successful case, like is there someone who joined the study and now is living without treatment.* |  |
|  |  | **05** | Partner with HIV | *I would like to ask something, as she has paused her treatment and she is on [trial] drugs. How will they see when the virus is no longer there?* |  |
| **Perceptions and Experiences during the ATI** | | | | | |
| **Understanding of ATIs** | | | | | |
| ATIs as part of research process | | **04** | Partner without HIV | *I will explain as it is, I will say someone who is on ATI, do not take their pills, it is a different kind of treatment… it is research coming from overseas, with big scientists.* |  |
|  |  | **01** | Sister | *They [researchers] are still trying to see if you can be able to control the virus which is still inside your body using the product while you are not on ARVs [ART]. It’s that during that time they still want to find out if your product does work… yes, I think it is that.* |  |
|  |  | **09** | Sister | *ATI – I would say that it is research that helps, about HIV to have a change so pills that can no longer be consumed… So that they will see if their research is working or not, in my own perspective.* |  |
| Hope for eventual cure | | **05** | Partner with HIV | *I will tell them that they can join this group because it had proved that they can evoke the virus, and fight it to a point that it is no longer there. And they could live without treatment forever.* |  |
|  |  | **08** | Partner without HIV | *I take it as a cure, I believe it will eliminate the virus… To see if ATI does work, that is all I can say.* |  |
|  |  | **06** | Mother | *To find cure medicine.* |  |
| Desire for clarity | | **02** | Cousin | *It is when treatment is being paused****.*** *It is where the treatment is being stopped to see if the reservoirs… Can you please remind me?* |  |
| Possible misunderstandings about ATIs | | **06** | Mother | *This one got an instruction from the doctor to pause her pills to see if the study drugs are working. She is the one on pause, you must continue with yours [treatment], they will see as time goes on if she will stay [on] pause or go back to her treatment. Even if she does not go back to treatment, that is okay, because they are the one [doctors/nurses] who know everything that is going on.* |  |
|  |  | **10** | Mother | *So it might happen when they no longer take their treatment, they will feel like they will die since they are defaulting. But when they were given the doses, a person understands that there is a time where they have to pause [ARVs] and life goes on and they will not have any problem.* |  |
| **Concerns for Trial Participants during ATI** | | | | | |
| Concerns about health risks | | **08** | Partner without HIV | *[I am worried] that she can have other illness like stroke, etc.* |  |
|  |  | **09** | Sister | *That since she has stopped taking pills, she will get sick, and it will be clear that she is like this, maybe she will have sores and symptoms.* |  |
| ATI-related concerns counterbalanced by clinical monitoring and support | | **03** | Partner without HIV | *One thing nice about research is whatever is happening, they first check if you are not at risk. So, every time she comes here to be checked, since she is on pause. She can be on pause, that is the purpose of the study. But if she come for checking to see if she is not at risk. She has told me that, there are other participants that are back on treatment. So, she is luck that she has not rebounded… What I understand is that research is a win or lose, but we cannot say it is a loss for us. We can say it is a lose to those who have rebounded… I do not have a worry… Since it is suppressed, I feel free about everything that is happening. Unless if she was not coming here to get monitored, I would have been worried because we will never know what will happen if she rebound. That would have been the problem, that is why I asked her today when was the last she came here to get checked. She said she comes every Wednesdays.* |  |
|  |  | **01** | Sister | *What concerns me is that, what if she encounters a problem while on ARV [ART] pause. But I have been told that as she comes here, she is being monitored and told what is right and wrong, and if it is found that something is wrong, she is then put back on and continues to use ARVs [ART].* |  |
|  |  | **02** | Cousin | *I have no concerns because I know that they are being checked before pausing them on ARVs [ART] and she also agreed. Before I came here, I checked with her if everything is still going alright, and she said that yes, everything is still going well. From what I know, since she has paused, she will continue to come here and be checked on her next visit and then see if she can still continue or go back to taking treatment. So, from what I have been told I am alright.* |  |
| **Concerns about the ATI from Partners** | | | | | |
| Partner with HIV: Risk of acquiring HIV | | **08** | Partner without HIV | *Yes, it is clear now that it is important to protect ourselves.* |  |
| Partners with HIV: Risks to their health from their partner’s HIV | | **07** | Partner with HIV | *Before all this started, I only saw risks... if she gets sick, I will also get sick.* |  |
| Partners with HIV: Risks of passing HIV to their partner during the ATI | | **05** | Partner with HIV | *Until she joined [the clinical trial], and she asked me that we use protection every time we are engaging in sexual intercourse. I asked her why because I still want family…So the time she is taking that drugs, I should not come and re-infect her with my virus [pass HIV again to her]. The pills she is getting, evoke the virus and then she gets another drug that fight the virus, so while fighting her virus, I should not come with mine and put her back to square one.* |  |
| Partner with HIV: Being blamed for “HIV treatment default” | | **07** | Partner with HIV | *It is that I feared that she will default, and her family will blame me as I am the one who is staying with her. That they will ask why I allowed her to pause her treatment, but then I knew I will tell them it is part of the study.* |  |
| Family members: no perceived risks to themselves from the ATI | | **10** | Mother | *No, I have not [seen any risk to myself].* |  |
|  |  | **01** | Sister | *No, I have never seen myself in any risk.* |  |
|  |  | **02** | Sister | *For now, I am alright, I do not foresee any danger.* |  |
| **HIV Transmission Risk during the ATI** | | | | | |
| **Partner Protections Plan during ATI** | | | | | |
| Partners without HIV | Awareness and understanding of HIV prevention options | **03** | Partner without HIV | *Yes, they did explain because I am welcome to come here [FRESH] and I now have contact number of some of the staff.* |  |
|  | Knowledge of PrEP and condoms | **04** | Partner without HIV | *I do think it is important to use both [PrEP] and condoms, because it decreases the risks.* |  |
|  | Behavioral adjustments  (e.g., abstinence) | **08** | Partner without HIV | *I feel it is best to abstain for that period she is given to pause her treatment… Yes, it is clear now that it is important to protect ourselves, or maybe we can abstain until she finishes that given period… I feel it is best to abstain for that period she is given to pause her treatment.* |  |
|  |  | **08** | Partner without HIV | *What I can do is continue using protection or minimize the number of times we engage in sexual intercourse, that’s all.* |  |
| Partners with HIV | Awareness and understanding of own HIV treatment options to remain undetectable | **07** | Partner with HIV | *Yes, she did tell me when she came from FRESH, she told me something has happened, her test results came positive. I told her I do not know how that happened, but we are both young, so she has her ways and I have my ways. But I am not saying there is something wrong she has done; we must find solution in all of this… I knew about her status first. So, with mind, I was already expecting that one day I will go for testing and find out what I already know. Because we continued to sleep together… It took a week, because I first went for testing without her knowledge, then on the second time I went with her. At the hospital they did explain that every individual has their own different system, for some people it takes time for the virus to be detected but at some stage it becomes detected… I went after 2 to 3 years then when I found out I told my dad, I have been found positive. He said the virus is something that you cannot guarantee that you will never have it, you can get it anywhere [from anybody] and you cannot blame your partner that she is the one who infected you. One thing you need to do, is to treat it, if you continue dating with her, you must make sure that you lead a normal life. This thing does not kill anymore, it only kills those who does not take care of themselves.* |  |
| **Additional Partner Protections Support** | | | | |  |
| Increased education on various HIV prevention options | | **04** | Partner without HIV | *To educate people, because what is important, is information. If you know, you can take care of yourself, because of the people they make decisions based on their emotions. They do not think but if they had information, you are able to think things thoroughly. Because from what I see PrEP and condoms are available, but people are lacking knowledge. Maybe if there can be a program where they will teach people about these things... Maybe there can be a program for learners in high school, maybe they will teach them in a different way compared to the adult group, the over 18.* |  |
|  |  | **04** | Partner without HIV | *The information should also be provided to partners. Perhaps teaching them is important, because some people don’t care about using condoms or simply don’t like them. So, having the right information can help protect them… I'm not sure if everyone is honest with their partners about this. Maybe you could explain it to the partner directly or provide pamphlets they can give to their partner. Alternatively, they could send information via WhatsApp or share videos with them.* |  |
|  |  | **04** | Partner without HIV | *Having access to platforms like WhatsApp would be beneficial, as people could send inquiries and receive valuable information directly. This approach would help educate people and empower them to take precautions, stay informed, and be more careful.* |  |
| **Social Support Needs** | | | | | |
| **Support Role during Clinical Trial** | | | | | |
| Commitment, care and support | | **03** | Partner without HIV | *I have already told myself that it is my duty to support her in life. So, she gets all the support in all aspects of life, there is nothing that I take as special as what she is doing here… I am here to support my partner.* |  |
|  |  | **08** | Partner without HIV | *What I can do for her is to check up on her, as she will be on pause of her treatment. And see if she is okay, and make sure that there are people around her who will help her if ever she faces some difficulties.* |  |
|  |  | **05** | Partner with HIV | *Yes, there is a role I can play, is to support her and take care of her in everything that she will need. I am able to come from work and cook, and she [can] relax.* |  |
|  |  | **06** | Mother | *Exactly, there were times where I told her to go and lie down, so that she can rest. Even with food, there were times where she will say, she feels like eating boiled food, because boiled food is healthy. If she doesn’t feel like oily food she can do boiled food, that is fine.* |  |
|  |  | **01** | Sister | *The support that I can give her is that all the time when she is needed here, she has to be here because she is not on ARVs if she doesn’t come, she won’t get support.* |  |
| Communication and relationship dynamics | | **04** | Partner without HIV | *I do not know, my role is support only, our relationship does not get changed by anything, we are still the same. We are friends, we talk about everything and anything… I will be able to help her, when I am off duty, I am able to help with taking care of the child and now she is not that stressed. We plan for our needs, when we need things like baby food, electricity we both cooperate. I feel like the pressure besides of the treatment, bNAb [broadly neutralizing antibodies] and ATI, besides of that, I can support her in life, and I think that is what important.* |  |
|  |  | **07** | Partner with HIV | *The role I played was to ask her if she is okay, as sometimes if I ask her, she will say she has a headache. My understanding is, she was not supposed to take any painkillers but Panado. So, my role is to tell her to call at FRESH if she is not feeling okay, I will tell her to ask them if she can take Panado or Grand-Pa [type of painkiller].* |  |
| Discovery and acceptance of HIV status | | **04** | Partner without HIV | *I was taken by her discipline, we dated, I visited her and I realized she respect herself. She can cook, take care of her child. So, I found out by myself about her status, she did not tell me. I found out in a different way. I saw her pills, but I was not sure, because she changed the container, but I noticed that, no these pills [suspicious]. But we have never talked about it, we continued dating, I wanted to wait for her to be ready to tell me. But I have realized that, that day will not come until we were expecting a child, and I asked her to tell me the truth because I know these pills… It was not a big issue, that I could not date her, because she is taking the treatment. I loved her, there was time where I just could not care if I get infected [acquire HIV], as long as we are together, I do not have a problem. But then I thought I must protect myself. Yes, I love her, but I have never judged her about her past. So, we continued dating, we still are, and I am enjoying the relationship… I saw her as someone disciplined, someone with integrity and someone who handles herself with dignity. Now I have found this, I am having doubts.* |  |
|  |  | **05** | Partner with HIV | *When I was diagnosed, I did not have an idea. I was having TB which I think disease [HIV] goes with TB. At that time, she was already at FRESH, so I went to test for TB and this disease [HIV] was also found. I took treatment for 6 months [TB treatment] and the one you take day and day [HIV treatment].* *[Interviewer: When did she know about her HIV status?] She was told here at FRESH, because she was already at FRESH, I can say we were both on the dark about our statuses. So, she joined FRESH, and she got to know her status, it is possible that she kept it a secret, considering our relationship was still new. I ended up coughing non-stop, and my chest had pain, so I went to a doctor. The doctor found TB and I was transferred to Mshiyeni [hospital], at Mshiyeni they found TB and this disease. So, we will never know, who came with this disease into the relationship between the both of us.* |  |
| Journey toward health and well-being | | **02** | Cousin | *The role I think I will play, is that I will see to it that she is doing things accordingly, because she is still young, she can still just decide to go out with her friends, and that is where I will come in and talk to her about such things.* |  |
|  |  | **09** | Sister | *To support her journey until the end… For her not to quit.* |  |
| **Additional Support Needed** | | | | | |
| Follow-up and home visits | | **08** | Partner without HIV | *They should support them by checking up on them even at their homes... if they miss their appointments, go and check them back home.* |  |
| Comprehensive support services | | **02** | Cousin | *Since I am her older sister, it has been my responsibility to make sure that she has the support she needs, but since she attends here, she is learning a lot which takes the burden away from me. She knows everything… they will need your support as much as we will be supporting them from home, so that they continue in this way.* |  |
| Social/educational activities or support groups | | **10** | Mother | *Uhm, how can I put it, because most of the time this group is attended mostly by females, I think that if they can group themselves and have an outing like doing a picnic, keep themselves busy, take themselves out, they must not always be on the same place [FRESH]. Meet others and hear their opinions… and hear from them how they feel as they are participating in the group; so that they can help people from around the world who do not like to take their [ARV] treatment.* |  |
| Joint involvement in clinical research | | **03** | Partner without HIV | *Well firstly I can say this program, it has just started, not so many people know about it. I only knew about it from her and other people who know it, are only those who come here. It would be better if it is not for women only [the program], if it can be open to everyone. I am sure many people would wish to be like her, because I am sure this has helped her a lot as much as it has helped me. Can you imagine if it was for everyone.* |  |
| **Additional Recommendations** | | | | |  |
| Desire for expanded education | | **08** | Partner without HIV | *No, there is not, besides that you have explained clearly everything to me. I now wish I can use PrEP to protect myself because I did have a clear understanding about it, that it prevents you from being infected [acquiring HIV].* |  |
| Close follow-up and monitoring for HIV cure trial participants | | **02** | Cousin | *I will recommend that they do the follow up to see how things are going…. I think in the way of checkups that they usually have.* |  |
| Communication and reminders | | **06** | Mother | *I have compliments because, they do write a date on their cards… let us say, their date is coming soon, they call to remind them. That is good because there are many things going through each person’s mind, that can make them to forget their clinic appointments.* |  |
| Emotional support | | **07** | Partner with HIV | *Yes, they are taking very good care of them, she always come back happy when she comes from here [FRESH]. Even if she was not well in her spirit but after going to FRESH, she will come back happy.* |  |
| Community and social connections | | **10** | Mother | *It encourages them, it makes them understand and admit that they are now living this life [with HIV].* |  |
